# Immune biomarkers to predict SARS-CoV-2 vaccine effectiveness in patients with hematological malignancies

**DOI:** 10.1038/s41408-021-00594-1

**Published:** 2021-12-14

**Authors:** Luis-Esteban Tamariz-Amador, Anna Martina Battaglia, Catarina Maia, Anastasiia Zherniakova, Camila Guerrero, Aintzane Zabaleta, Leire Burgos, Cirino Botta, Maria-Antonia Fortuño, Carlos Grande, Andrea Manubens, Jose-Maria Arguiñano, Clara Gomez, Ernesto Perez-Persona, Iñigo Olazabal, Itziar Oiartzabal, Carlos Panizo, Felipe Prosper, Jesus F. San-Miguel, Paula Rodriguez-Otero, Esperanza Martín-Sánchez, Bruno Paiva, The Asociación Vasco-Navarra de Hematología y Hemoterapia (ASOVASNA) Cooperative Group

**Affiliations:** 1 Clinica Universidad de Navarra, Centro de Investigacion Medica Aplicada (CIMA), IDISNA, CIBERONC, Pamplona, Spain; 2 Department of Experimental and Clinical Medicine, “Magna Graecia”, University of Catanzaro, Catanzaro, Italy; 3 Russian Research Institute of Hematology and Transfusiology, Saint-Petersburg, Russian Federation; 4 Complejo Hospitalario de Navarra, IDISNA, Pamplona, Spain; 5 Hospital Universitario de Galdakao, Galdakano, Spain; 6 Hospital Universitario de Araba – sede Txagorritxu, Vitoria, Spain; 7 Hospital Universitario de Donostia, San Sebastian, Spain; 8 Hospital Universitario de Cruces, Bilbao, Spain

**Keywords:** Haematological cancer, Tumour immunology

## Abstract

There is evidence of reduced SARS-CoV-2 vaccine effectiveness in patients with hematological malignancies. We hypothesized that tumor and treatment-related immunosuppression can be depicted in peripheral blood, and that immune profiling prior to vaccination can help predict immunogenicity. We performed a comprehensive immunological characterization of 83 hematological patients before vaccination and measured IgM, IgG, and IgA antibody response to four viral antigens at day +7 after second-dose COVID-19 vaccination using multidimensional and computational flow cytometry. Health care practitioners of similar age were the control group (*n* = 102). Forty-four out of 59 immune cell types were significantly altered in patients; those with monoclonal gammopathies showed greater immunosuppression than patients with B-cell disorders and Hodgkin lymphoma. Immune dysregulation emerged before treatment, peaked while on-therapy, and did not return to normalcy after stopping treatment. We identified an immunotype that was significantly associated with poor antibody response and uncovered that the frequency of neutrophils, classical monocytes, CD4, and CD8 effector memory CD127low T cells, as well as naive CD21+ and IgM+D+ memory B cells, were independently associated with immunogenicity. Thus, we provide novel immune biomarkers to predict COVID-19 vaccine effectiveness in hematological patients, which are complementary to treatment-related factors and may help tailoring possible vaccine boosters.

## Introduction

The novel coronavirus disease 2019 (COVID-19) pandemic has spread throughout the world, with over 249 million confirmed cases globally and more than 5 million deaths as of November 2021 (https://covid19.who.int/). Compared with the general population, cancer patients are at greater risk of serious COVID-19-related complications and fatal outcome [1, 2]. Higher mortality was observed in hematological patients when compared to those with solid tumors [3–6], which likely reflects immune impairment from the underlying blood cancer as well as therapies that disable innate, B, and T-cell immunity [5, 7–10]. According to large studies, nearly one-third of individuals with hematological malignancies have died with COVID-19 [11–13]. Thus, effective vaccination against severe acute respiratory syndrome coronavirus 2 (SARS-CoV-2) is of utmost importance to prevent COVID-19 in these patients.

There was great concern that individuals with cancer may not mount a robust protective immune response to SARS-CoV-2 vaccination and initial reports investigating vaccine effectiveness in this population showed that antibody production was more likely to occur in patients with solid tumors than in those with hematological malignancies [14–16]. Subsequent studies across the spectrum of hematological malignancies uncovered remarkable heterogeneity in response to vaccination, with the poorest rates of seropositivity being generally observed in patients with B-cell chronic lymphocytic leukemia (CLL), non-Hodgkin lymphoma, and multiple myeloma (MM) [17]. By contrast, seroconversion was higher in cases with acute leukemia, chronic myeloid leukemia, myelodysplastic syndromes, and Hodgkin lymphoma [18, 19]. After these initial observations, larger and disease-specific studies in patients with B-cell and plasma cell (PC) disorders were most recently reported.

The efficacy of SARS-CoV-2 vaccine in CLL patients was 39.5% and 75% in two large Israeli and UK studies [20, 21]. In both cohorts, increased rates of seronegativity were associated with immunoparesis and the use of Bruton’s tyrosine kinase inhibitors. By contrast, the negative impact of venetoclax and anti-CD20 therapy was not consistent. In patients with B-cell lymphoma, seronegativity was found to be associated with indolent vs. aggressive subtypes and with B-cell suppressive therapies [18, 22, 23]. Nonetheless, poor serological responses were observed in some patients who did not receive therapy in the past 2 years [18]. In MM, clinically relevant antibody responses were generally observed in ≥50% of patients [18, 24–30]. Although suboptimal seroconversion was common on active treatment, discordant results on the impact of anti-CD38 and anti-BCMA antibodies, as well as steroids were reported [26–28, 30].

The great heterogeneity observed in the efficacy of vaccines for COVID-19 among hematological patients is probably associated with both therapy-related immunosuppression and disease-related immune dysregulation. Thus, we conducted a prospective study to determine the immune landscape of patients with a mature B-cell and PC neoplasm prior vaccination and its relationship with antibody response after two doses of SARS-CoV-2 vaccine. We hypothesized that immune profiling prior vaccination could be complementary to other clinical features in the identification of hematological patients at risk of weak immunogenicity.

## Patients and methods

### Study design

**Fig. 1:**
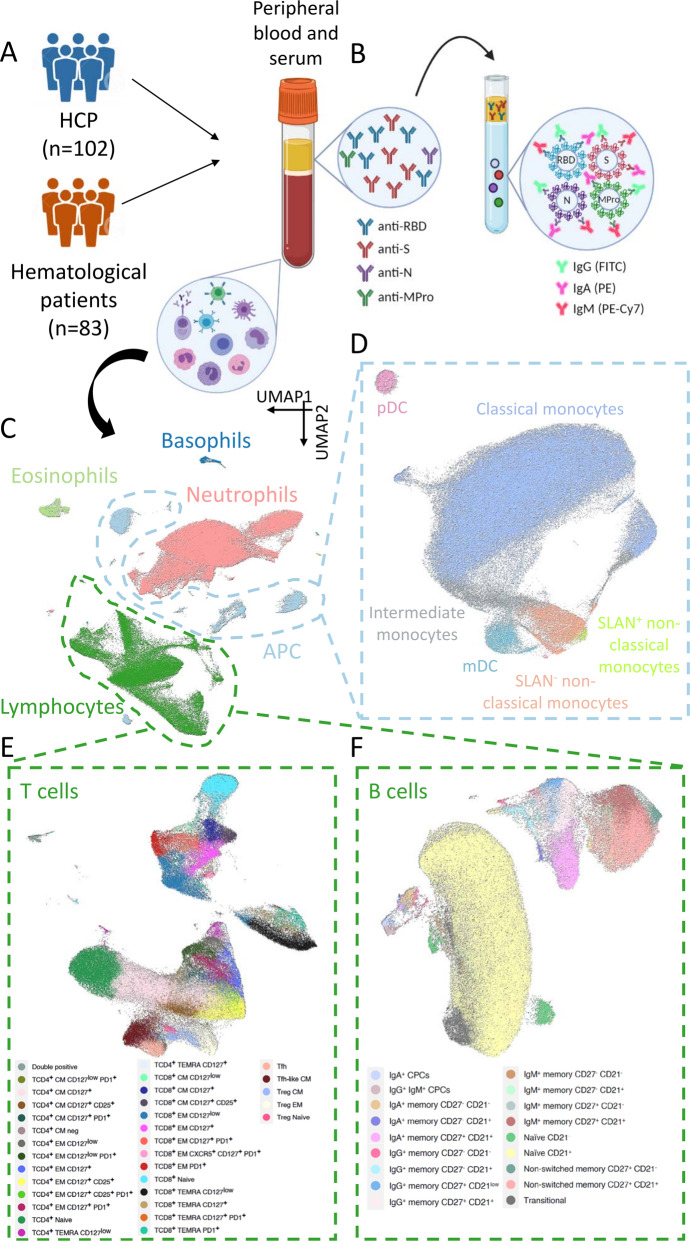
Study design. **A** Peripheral blood and serum from 83 patients with hematological malignancies and 102 health care practitioners (HCP) were analyzed. **B** Antibody response at day 7 after the second-dose vaccination was measured using a CE-IVD serological SARS-CoV-2 multiplex bead-based flow cytometry immunoassay. It allows the simultaneous and quantitative detection of specific IgM, IgG, and IgA antibodies to four different antigens present in serum: (1) the receptor-binding domain (RBD) of the S-glycoprotein; (2) the stable trimer of the spicule (S) glycoprotein; (3) the nucleocapsid (N) protein; and (4) the main virus protease (Mpro). Detection of antibodies against the N and Mpro antigens allows the identification of individuals infected with SARS-CoV-2 prior or during vaccination. **C** Immune profiling of hematological patients and HCP prior vaccination was performed using multidimensional and computational flow cytometry. A total of 59 immune cell types were systematically measured in peripheral blood, including basophils, eosinophils, neutrophils, antigen-presenting cells (APC) and lymphocytes. **D** APC were sub-clustered into classical, intermediate, SLAN− and SLAN+ non-classical monocytes, as well as plasmacytoid and myeloid dendritic cells (pDC and mDC, respectively). **E** Sub-clustering of T cells into 32 subsets related to antigen-dependent differentiation, as well as activation and exhaustion phenotypes in helper and cytotoxic compartments. **F** Sub-clustering of B cells into 18 subsets related to antigen-dependent differentiation. CM, central memory; EM, effector memory; TEMRA, effector memory T cells re-expressing CD45RA; Tfh, follicular helper T cells; Treg, regulatory T cells; CPCs, circulating plasma cells.

A total of 83 patients with hematological malignancies and 102 health care practitioners (HCPs), vaccinated between January and June 2021, were studied (Fig. 1A). Only HCP older than 50 were included as the control group to match the expected age of patients. All subjects had no documented SARS-CoV-2 infection. Peripheral blood and serum were collected prior to the first dose, at days 7 and 14 after the first dose, at day 7 after the second-dose and at day 100 after the first-dose vaccination. The study was approved by the Ethics Committee of the Comunidad Foral de Navarra (2021.006) and was conducted per the ethical principles of the Declaration of Helsinki. All HCP and patients provided written informed consent prior enrollment in the study. Samples and data from subjects included in the study were provided by the Biobank of the University of Navarra and were processed following standard operating procedures approved by the Ethical and Scientific Committees. No data were collected regarding vaccination side effects or toxicities, as this was outside the scope of the study.

### Assessment of serological response

Antibody response at day 7 after second-dose vaccination and at day 100 after first-dose vaccination (in 100 HCP and 79 patients) was assessed using a CE-IVD serological SARS-CoV-2 multiplex bead-based flow cytometry immunoassay (Immunostep SL, Salamanca, Spain) (Fig. 1B) [31]. It allows the simultaneous and quantitative detection of specific IgM, IgG, and IgA antibodies to four different viral antigens present in serum: (1) the receptor-binding domain (RBD) of the S-glycoprotein; (2) the stable trimer of the spicule (S) glycoprotein; (3) the nucleocapsid (N) protein; and (4) the main virus protease (Mpro). Detection of antibodies against the N and Mpro antigens allows the identification of individuals infected with SARS-CoV-2 before or during vaccination. Thus, the assay enabled the accurate discrimination between vaccinated and naturally infected individuals (i.e., negative and positive for both antigens, respectively).

First, samples were diluted (1 : 20 and 1 : 200 to respectively measure IgM/IgA and IgG antibodies in HCP; 1 : 10 and 1 : 100 to respectively measure IgM/IgA and IgG antibodies in patients) before incubation with beads, at which stage there is binding of antigen-specific antibodies to the respective proteins that are coating the beads. Each bead has a unique fluorescence intensity pattern and is coated with one of the SARS-CoV-2 antigens enumerated above (RBD, S, N, and Mpro). After washing to remove unbound antibodies, fluorophore-conjugated anti-human IgM (PE-Cy7), IgG (fluorescein isothiocyanate, FITC), and IgA (PE) were added to quantify antigen-specific antibodies bound to beads. After another washing step, beads were suspended and the fluorescence intensity was measured in the PE-Cy7, FITC, and PE channels, which is proportional to the amount of IgM, IgG, and IgA antibodies present in serum, respectively. Samples were analyzed in a CytoFlex LX (Beckman Coulter [BC] Life Sciences, IN) using the CytExpert v2.3 software (BC) and in an Aurora (Cytek Biosciences, CA) using the SpectroFlo v3.0.0 software (Cytek). Antibody indexes were calculated following the manufacturer’s formula [(mean fluorescence intensity of the sample/mean fluorescence intensity of the negative control provided in the kit) × 10] and detection of antibodies was considered positive if antibody indexes were ≥12. The assay is calibrated following the WHO First International Standard for Anti-SARS-CoV-2 Human Immunoglobulin (NIBSC Code 20/136). Thus, we reported the concentration of IgG in international units (IU/mL) against RBD or in binding antibody units (BAU/mL) against S viral antigens. The dilution range to obtain a lineal regression model (*r* ≥ 0.99) was of 1 : 8 to 1 : 16,384.

### Immune profiling

Peripheral blood was collected in EDTA containing tubes and processed within 24 h following the EuroFlow lyse-wash-and-stain protocol (adjusted to 10^6^ nucleated cells) [32]. Eight-color monoclonal antibody combinations (Supplemental Table 1) were developed to enumerate major lymphocyte and myeloid compartments (Fig. 1C) and characterize the relative distribution of granulocytes, antigen-presenting cells (APCs), T-cell, and B-cell subsets within each of these compartments (Fig. 1C–E). Overall, 59 immune cell types were systematically evaluated in peripheral blood of hematological patients and HCP (Supplemental Fig. 1 and Supplemental Table 2). Samples were measured in a FACSLyric flow cytometer (Beckton Dickinson Biosciences [BD], San Jose, CA, USA) using FACSuite v1.3.0.6137 software (BD).

### Computational flow cytometry analysis

Data were analyzed using *FlowCT*, a semi-automated workflow developed for deconvolution of immunophenotypic data and objective reporting on large datasets [33]. Briefly, *FlowCT* starts by creating a matrix with expression data generating a SingleCellExperiment object and correcting possible discrepancies in markers’ nomenclature. Subsequently, it performs internal data quality control and normalization, and automated clustering followed by dimensionality reduction to visualize clusters’ identity before manual annotation. This step is completed using the Infinicyt software (Infinicyt v2.0; Cytognos SL, Salamanca, Spain). Afterwards, sub-clustering of APC, T, and B cells was performed. Comma-separated value files with population abundances were finally exported to evaluate statistical correlations and differences across groups.

### Statistical analysis

Immune profiles and seroconversion were compared between HCP vs. patients, and between groups of patients using proportions of immune cell types and antibody indexes or concentrations. The Kruskal–Wallis and Mann–Whitney tests were performed to estimate the statistical significance observed between groups and the *χ*^2^ and Mann–Whitney *U*-tests were performed to test distributions between immunotypes resulting from unsupervised clustering. Logistic regression was performed to analyze the relationship between clinical and immune covariates in predicting antibody response to SARS-CoV-2 vaccination (defined as ≥553.5 IU/mL anti-RBD IgG) in hematological patients. First, cutoffs were calculated for the 59 immune cell types to maximize the area under the receiver operating characteristics curve (AUC). *χ*^2^ and odds ratio univariate analyses were subsequently performed to filter variables associated with antibody response (*P* < 0.1). Further feature selection for immune covariates was performed using lasso regularization for logistic regression (scikit-learn), where coefficients of nonsignificant or multi-colinear variables are shrunk to 0. A logistic regression machine learning model was generated for six immune and two clinical covariates and evaluated using fourfold cross-validation.

All statistical analyses were performed using the GraphPad Prism (version 7, San Diego, CA, USA), SPSS (version 25.0.0, IBM, Chicago, IL, USA), and R (version 4.0.0) software. Machine learning analyses were performed using Python (version 3.8.6) and scikit-learn (version 0.23.2). *P*-values of <0.05 were considered statistically significant.

## Results

### Characteristics of hematological patients and HCP

The median age of patients and HCP was 55 (Table 1). There was an increased proportion of female HCP, whereas other variables such as body mass index > 30 and comorbidities such diabetes, hypertension, and autoimmune disease were generally balanced between patients and HCP. All but one patient and seven HCP received mRNA SARS-CoV-2 vaccines; 59% of patients were administered BNT162b2, whereas 66% of HCP were vaccinated with mRNA-1273 (Table 1).

**Table 1:** Characteristics and type of vaccine for COVID-19 administered to health care practitioners (HCPs) and patients with hematological malignancies.

Characteristics	HCP (*N* = 102)	Patients (*N* = 83)
Age, median (range), years	55 (50–69)	55 (21–85)
Male, no. (%)	10 (10%)	45 (54%)
Comorbidities, no. (%)		
Any	25 (24.5%)	21 (25%)
Body mass index > 30	11 (11%)	13 (16%)
Diabetes	0 (0%)	7 (8%)
Hypertension	8 (8%)	11 (13%)
Autoimmune disease	7 (7%)	3 (4%)
Hematological malignancy, no. (%)		
B-cell lymphoproliferative disorders	-	48 (58%)
Monoclonal gammopathies	-	28 (34%)
Hodgkin lymphoma	-	7 (8%)
Type of vaccine, no. (%)		
BNT162b2	28 (27.4%)	49 (59.0%)
mRNA-1273	67 (65.7%)	33 (39.8%)
ChAdOx1	7 (6.9%)	1 (1.2%)

Of the 83 patients enrolled, seven had Hodgkin lymphoma and the remaining 76 had a mature B-cell or PC neoplasm (Table 2). There were seven cases diagnosed with CLL, 25 with indolent and 16 with aggressive non-Hodgkin lymphoma subtypes and 28 patients had a monoclonal gammopathy (one monoclonal gammopathy of undetermined significance, two smoldering and 25 active MM). The median time from diagnosis was 3 years (range, 0.2–26). Although the scope of the study was in patients with a mature B-cell or PC neoplasm, those with Hodgkin lymphoma were not excluded from the study population due to less information on the vaccine effectiveness in this disease. In addition, it allowed the comparison of immune profiles in patients with Hodgkin lymphoma vs. patients with a B-cell lymphoproliferative disorder or a monoclonal gammopathy.

**Table 2:** Seroconversion in 83 patients with hematological malignancies.

Disease	Patients
B-cell lymphoproliferative disorders	36/48
Chronic lymphocytic leukemia	4/7
Indolent non-Hodgkin lymphoma	18/25
Follicular lymphoma	10/14
Marginal zone lymphoma	4/6
MALT lymphoma	0/1
Lymphoplasmacytic lymphoma	3/3
Hairy-cell leukemia	1/1
Aggressive non-Hodgkin lymphoma	14/16
Diffuse large B-cell lymphoma	9/10
Cutaneous B-cell lymphoma	2/3
Burkitt lymphoma	2/2
Mediastinal B-cell lymphoma	1/1
Monoclonal gammopathies	17/28
MGUS	1/1
Smoldering multiple myeloma	2/2
Active multiple myeloma	14/25
Hodgkin lymphoma	6/7
Treatment	
Never received	14/17
On	6/17
Off	39/49
Lines of therapy	
1	35/44
2 or more	10/22
Previous treatment	
Anti-CD20	22/27
Anti-CD38	6/12
Immunomodulatory drugs	11/22
Autologous stem cell transplantation	14/20
Depth of response before vaccination in treated patients	
Complete response	54/66
No complete response	12/66

At the time of vaccination, 17 (20.5%) patients had never received anti-tumor treatment, 17 (20.5%) were on-therapy, and 49 (59%) were off. The median number (and range) of lines in patients on and off-treatment was ﻿2 (1–4) and 1 (1–5), respectively. Among treated patients, 54/66 (82%) were in complete response (CR) and 12/66 (18%) in less than CR (Table 2).

### Immune landscape of hematological patients and HCP

**Fig. 2:**
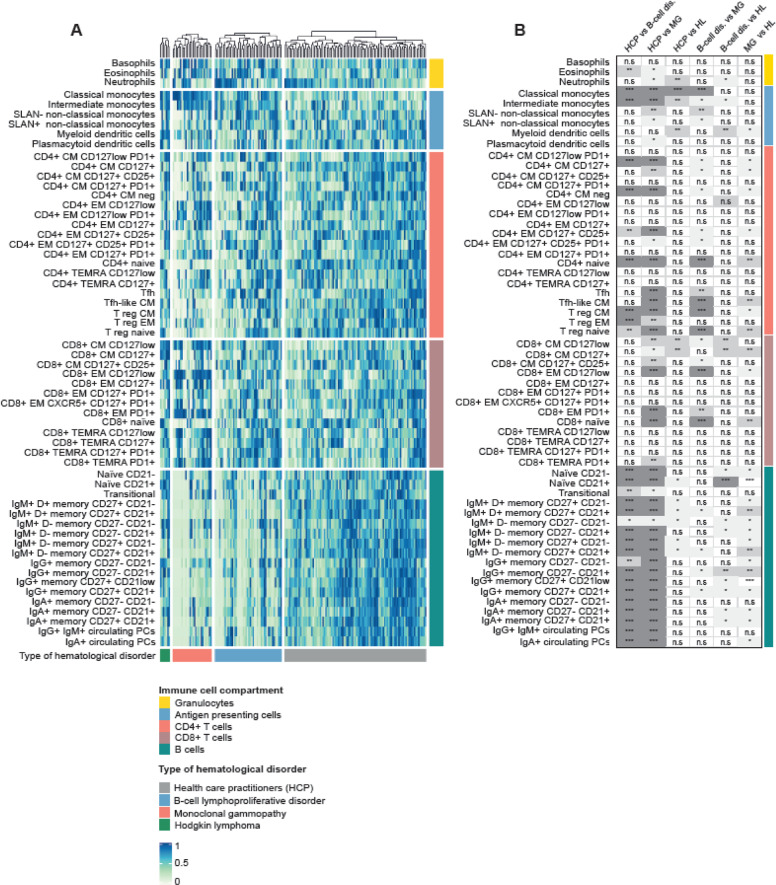
The immune landscape of hematological patients and HCP. **A** Supervised clustering of 83 patients with hematological malignancies and 102 health care practitioners (HCP), based on the percentile distribution of 59 immune cell types in peripheral blood that were categorized into five groups: granulocytes, antigen-presenting cells (APC), CD4, and CD8 T cells and B cells. Patients were grouped based on the diagnosis of a B-cell lymphoproliferative disorder (B-cell dis.), monoclonal gammopathies (MG), and Hodgkin lymphoma (HL). **B** Statistical significance of differences across groups (with graphical representation of such differences in Supplemental Fig. 2). CM, central memory; EM, effector memory; Treg, T regulatory cells; Tfh, T follicular-like; TEMRA, terminally effector memory CD45RA+; PC, plasma cells. *P < 0.05; **P < 0.01; ***P < 0.001; n.s., not significant.

When compared to HCP, 44 of the 59 (75%) immune cell types evaluated in peripheral blood were significantly altered in patients belonging to at least one of the three main disease categories: B-cell lymphoproliferative disorders (28/59, 47%), monoclonal gammopathies (43/59, 73%), and Hodgkin lymphoma (12/59, 20%). The B-cell compartment was similarly skewed in patients with a B-cell and a PC neoplasm vs. HCP. Those with monoclonal gammopathies showed additional redistribution of CD4 and CD8 T-cell subsets, as well as of APC (Fig. 2 and Supplemental Fig. 2). All three regulatory T (Treg) cell types identified in this study were significantly reduced in cases with a B-cell and PC neoplasm; two follicular helper (Tfh) cell types were additionally skewed in those with monoclonal gammopathies. Patients with Hodgkin lymphoma displayed mild deregulation of the B- and T-cell compartments, and the most significant alterations were observed in APC. Interestingly, a significant increment of classical and intermediate monocytes was found across hematological patients.

### Tumor-related immune dysregulation and therapy-related immunosuppression

**Fig. 3:**
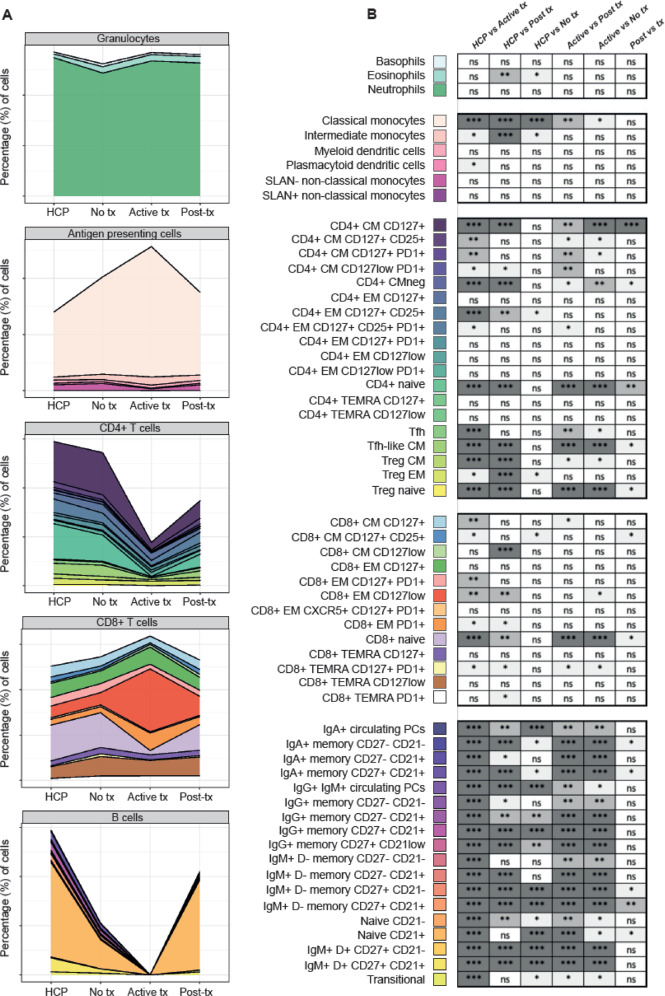
Tumor-related immune dysregulation and therapy-related immunosuppression. **A** Relative distribution of granulocytes, antigen-presenting cells (APC), CD4 and CD8 T cells, and B cells across health care practitioners (HCPs), hematological patients that had never received treatment (No tx, *N* = 17), those that were on (Active tx, *N* = 17), and patients that were off-treatment (Post-tx, *N* = 49) before vaccination. **B** Statistical significance of differences across groups (with graphical representation of such differences in Supplemental Fig. 3). APC, antigen-presenting cells; CM, central memory; EM, effector memory; Treg, T regulatory cells; Tfh, T follicular-like; TEMRA, terminally effector memory CD45RA+; PC, plasma cells. *P < 0.05; **P < 0.01; ***P < 0.001; n.s., not significant.

Next, we analyzed the immune status of HCP compared to that of patients that had never received anticancer therapy (i.e., tumor-related immune dysregulation), were on-treatment or had finalized their regimen (i.e., therapy-related immunosuppression). When comparing the relative distribution of the 59 immune cell types to that of HCP, patients that were on and off-treatment showed more frequent alterations than those that were never treated (39/59 [66%], 33/59 [56%], and 20/59 [34%], respectively). An increased frequency of classical and intermediate monocytes was, once more, found across hematological patients, whereas other granulocytic cell types and APC were less altered (Fig. 3 and Supplemental Fig. 3). The antigen-dependent differentiation of CD4 and CD8 T cells was frequently altered in patients on and off-treatment, whereas it was similar between HCP and patients that were never treated. The most significant differences within the T-cell compartment were observed in CD4 and CD8 naive and central memory subsets, as well as in Treg and Tfh cell types. Alterations found in 13 out of the 18 B-cell subsets analyzed in this study were observed in patients that were never treated, peaked to 18/18 in those on active therapy, and did not bounce back once treatment finalized (15/18).

### Antibody response in hematological patients and HCP

**Fig. 4:**
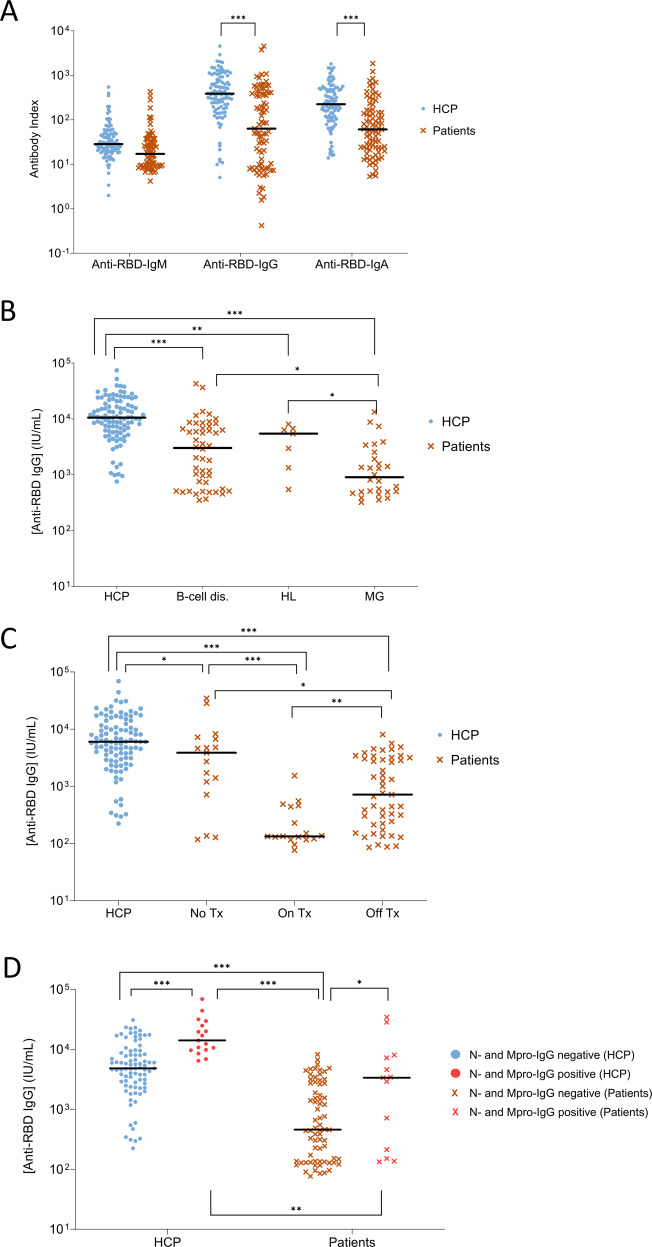
Antibody response in hematological patients and HCP. **A** Index of IgM, IgG, and IgA antibodies against the receptor-binding domain (RBD) of the S-glycoprotein in health care practitioners (HCP, *N* = 102) and hematological patients (*N* = 83). **B** Concentration of anti-RBD IgG in HCP, hematological patients with B-cell lymphoproliferative disorders (B-cell dis., *N* = 48), monoclonal gammopathies (MG, *N* = 28) and Hodgkin lymphoma (HL, *N* = 7). **C** Concentration of anti-RBD IgG in HCP, hematological patients that had never received treatment (No Tx, *N* = 17), those that were on (*N* = 17), and patients that were off-treatment (*N* = 49) before vaccination. **D** Concentration of anti-RBD IgG in HCP and hematological patients with or without previous SARS-CoV-2 infection, based on the detection of IgG antibodies against the nucleocapsid (N) protein and the main virus protease (Mpro). Among HCP, 85 were negative and 17 positive for both antigens. Among hematological patients, 70 were negative and 13 positive for both antigens. In all panels, horizontal lines represent the median. *P < 0.05; **P < 0.01; ***P < 0.001.

Based on the antibody index of IgG against the RBD antigen (which is the major target for virus-neutralizing antibodies [34]), the percentage of patients that tested positive at day 7 after the second-dose vaccination was significantly inferior than that of HCP (59/83 [71%] vs. 98/102 [96%], respectively; *P* < 0.001). Furthermore, the antibody indexes of IgG and IgA against the RBD antigen were significantly lower in patients vs. HCP (Fig. 4A). Similar results were observed for anti-S antibody response (Supplemental Fig. 4).

Seropositivity was more frequent in patients with Hodgkin lymphoma (6/7, 86%) and B-cell lymphoproliferative disorders (36/48, 75%) when compared to those with monoclonal gammopathies (17/28, 61%) (Table 2). Seroconversion rates were greater in patients who never received treatment (14/17, 82%), dropped considerably in those on-therapy (6/17, 35%), and bounced back once patients were off-treatment (39/49, 80%). Furthermore, antibody detection was more frequent in patients who received one (35/44, 80%) vs. two or more lines of therapy (10/22, 45%). Previous treatment with anti-CD38 monoclonal antibodies reduced to half the chances of immunogenicity (6/12, 50%), particularly in patients that were on active treatment. The negative impact of autologous transplant (14/20, 70%) and anti-CD20 therapy (22/27, 81%) in seroconversion rates was less pronounced. Patients in complete remission (40/54, 70%) were more frequently seropositive than those in other response categories (19/29, 66%).

Further analyses of antibody titers according to international units showed that hematological patients had significantly reduced anti-RBD IgG (median 553 vs. 6014 IU/mL; *P* < 0.001) and anti-S IgG (median 912 vs. 5313 BAU/mL; *P* < 0.001) compared to HCP. Median anti-RBD IgG levels were of 1262, 279, and 2634 IU/mL in patients with B-cell lymphoproliferative disorders, monoclonal gammopathies, and Hodgkin lymphoma, respectively. Thus, patients with a PC disorder had significantly lower anti-RBD IgG titers when compared to the other two disease categories (Fig. 4B). Median anti-RBD IgG levels were systematically higher in HCP vs. patients, regardless of whether the latter had never been treated or if they were on or off-therapy (3871, 133, and 719 IU/mL, respectively). Notwithstanding, the concentration of anti-RBD IgG was significantly lower in patients on active treatment vs. those that were off (*P* = 0.001) or had never received anti-tumor therapy *(P* < 0.001*)* (Fig. 4C). Similar results were observed for anti-S IgG titers (data not shown). Patients vaccinated with mRNA-1273 displayed significantly higher anti-RBD IgG levels than those vaccinated with BNT162b2 (1535.64 vs. 412.88 IU/mL, respectively; *P* = 0.016). No differences were observed in HCP (Supplemental Fig. 5). As expected, anti-RBD IgG titers significantly decreased from day 7 after second-dose to day 100 after first-dose vaccination in patients (553–249 IU/mL, *P* < 0.001) and HCP (6014–355 IU/mL, *P* < 0.001).

### Undocumented SARS-CoV-2 infection in hematological patients and HCP

Seventeen of the 102 (17%) HCP and 13/83 (16%) patients tested positive for the N and Mpro virus-specific antigens, and therefore were unaware of being infected with SARS-CoV-2 before or during vaccination. As expected, anti-RBD IgG titers were significantly higher in HCP previously exposed to SARS-CoV-2 when compared to those who were not (median 14,104 vs. 4817 IU/mL, respectively), and a similar kinetics was observed in patients (median 3368 vs. 458 IU/mL, respectively). Interestingly, the fold-change in anti-RBD IgG levels between previously infected and non-infected patients was higher than that observed in HCP (seven- vs. threefold, respectively), without statistically significant differences in the median concentration of anti-RBD IgG after two doses of SARS-CoV-2 vaccine between previously infected patients and non-infected HCP (Fig. 4D). There were no statistical differences in the distribution of the 59 immune cell types in HCP with or without previous infection, and the same applied for the comparison between patients.

### Immunotypes associated with poor antibody response

After observing altered immune profiles prior to vaccination, and lower seroconversion rates and reduced antibody production after vaccine administration in patients vs. HCP, we next aimed to perform a holistic analysis of patients’ demographics and disease features, treatment, and immune landscape, in relation to seroconversion and the generation of relevant concentrations of anti-RBD IgG antibodies. To this end, we stratified patients according to titer levels: lower or >553.5 IU/mL. This cutoff was selected because it represented the median concentration of anti-RBD IgG found in the 83 hematological patients and because it segregated most HCP (95/102 [93%]) from a few with clearly lower titers (Supplemental Fig. 6). Vaccine effectiveness in reducing the incidence of symptomatic and asymptomatic SARS-CoV-2 infection was reported to be >90% in HCP [35–37]. In fact, in our cohort only 4/102 (4%) developed (mild) COVID-19 after vaccination. Thus, it can be inferred that the presence of ≥553.5 IU/mL anti-RBD IgG confers protection to COVID-19.

**Fig. 5:**
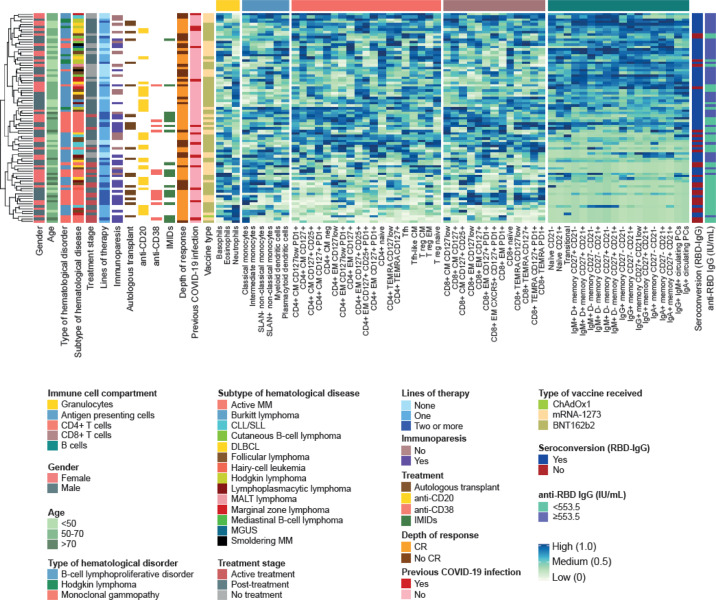
Immunotypes associated with poor antibody response. Unsupervised clustering of 83 hematological patients and 102 health care practitioners (HCPs) based on the relative percentile distribution of 59 immune cell types in peripheral blood before vaccination, categorized into four groups: granulocytes, antigen-presenting cell (APC) subsets, T-cell and B-cell subsets. For the columns to the left of the cell-percentage data, moving from left to right, rows are color-coded according to gender, age groups, type and subtype of hematological malignancy, treatment status, number of previous lines of therapy, immunoparesis, autologous transplant, treatment with anti-CD20 antibodies, anti-CD38 antibodies and immunomodulatory drugs (IMiDs), depth of response to treatment (complete remission, CR), previous SARS-CoV-2 infection, and vaccine type. For the columns to the right of the cell-percentage data, moving from left to right, rows are color-coded according to seroconversion and presence of ≥553.5 IU/mL IgG against the receptor-binding domain (RBD) of the S-glycoprotein. CM, central memory; EM, effector memory; Treg, T regulatory cells; Tfh, T follicular-like; TEMRA, terminally effector memory CD45RA+; PC, plasma cells.

The first two branches after unsupervised clustering analysis according to the relative distribution of the 59 immune cell types, divided the 83 hematological patients in 2 groups: 1 with 55 (66%) and another, smaller and more homogeneous with 28 (34%) patients (Fig. 5). When compared to the larger branch, the smallest was characterized by lower seroconversion rates (8/28 [29%] vs. 45/55 [82%], *P* = 0.006) and <553.5 IU/mL anti-RBD IgG (7/28 [25%] vs. 35/45 [78%], *P* = 0.002). Half of the patients in the smallest branch had a B-cell lymphoproliferative disorder and the other half, a monoclonal gammopathy; by contrast, all cases with Hodgkin lymphoma clustered in the larger branch (*P* = 0.03). Patients belonging to the smaller branch were older (*P* = 0.007) and had received treatment more frequently (*P* = 0.002) including ≥2 lines of therapy (*P* = 0.018). Treatment with anti-CD38 antibodies (*P* = 0.02) and immunomodulatory drugs ([IMiDs], *P* = 0.0099) was more frequent in these patients as well. Gender, exposure to anti-CD20 or autologous transplant, the type of vaccine, and previous SARS-CoV-2 infection were not significantly associated with patients’ branch.

The two branches were characterized by altered distribution of granulocytes, APC, CD4 and CD8 T cells, as well as B cells (Fig. 5 and Supplemental Fig. 7). Neutrophils and classical monocytes were significantly increased in patients belonging to the smaller branch, whereas SLAN+ non-classical monocytes were reduced. Within the T-cell compartment, the frequency of 17/19 CD4 subsets and of 8/13 CD8 subsets were significantly decreased in patients belonging to the smaller branch. All 18 subsets of the B-cell compartment were markedly reduced in these cases. Overall, this holistic analysis uncovered the presence of a distinct immunotype that was significantly associated with weak immunogenicity, as well as patients’ age, disease category, treatment status, and previous exposure to anti-CD38 antibodies and IMiDs. As these two agents were frequently administered in the same patients, either simultaneously or in different lines, the isolated effect of each drug cannot be determined.

### Immune biomarkers of immunogenicity

Optimal cutoffs for association with the generation of relevant concentrations of anti-RBD IgG antibodies were determined for percentages of each of the 59 immune cell types based on their AUC (Supplemental Table 3). Using these cutoffs, the frequencies of 43 immune cell types were significantly associated with less or >553.5 IU/mL anti-RBD IgG. Lasso regression was subsequently performed for feature selection, and six immune cell types were selected by the algorithm for logistic regression multivariate analysis: neutrophils, classical monocytes, CD4 and CD8 effector memory CD127low T cells, as well as naive CD21+ and IgM+D+ memory B cells. Clinical parameters significantly associated with < or >553.5 IU/mL anti-RBD IgG were the diagnosis of a monoclonal gammopathy (*P* = 0.03), treatment status (*P* < 0.001), number of prior lines of therapy (*P* = 0.003), and anti-CD38 therapy (*P* = 0.03). Diagnosis of a monoclonal gammopathy decreased the model’s accuracy and was therefore excluded from the logistic regression. Other patient-related or disease-related characteristics, including age and treatment with IMiDs, were not significantly associated.

**Fig. 6:**
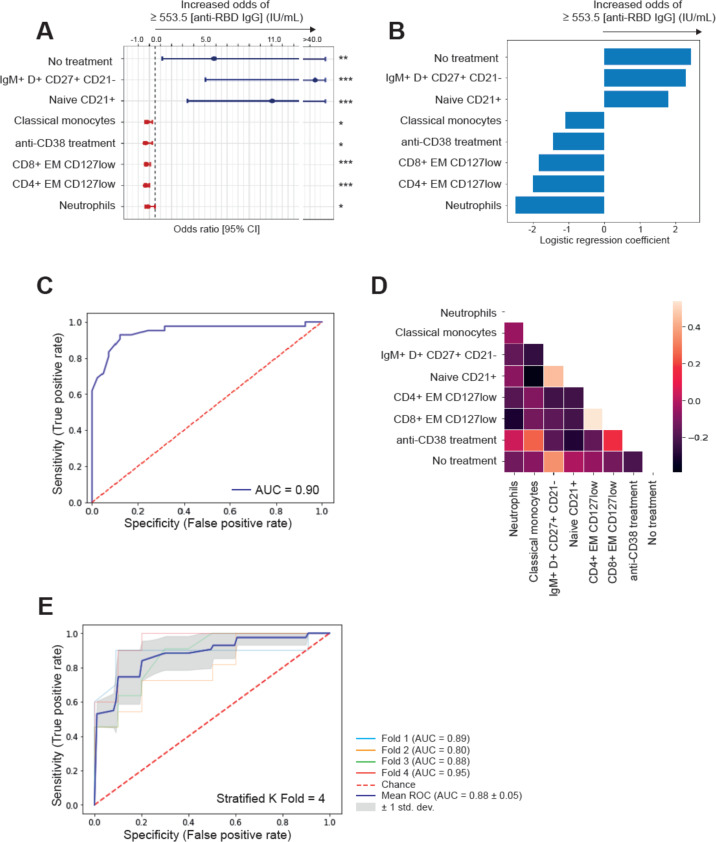
Immune biomarkers of immunogenicity. **A** Odds ratio univariate analysis with 95% confidence intervals (CI) of variables included in the logistic regression model. **B** Logistic regression coefficients with treatment-related features as well as immune biomarkers associated with generation of ≥553.5 IU/mL of IgG antibodies against the receptor-binding domain (RBD) of the S-glycoprotein. **C** Area under the curve (AUC) of prediction probabilities of patients**’** dataset. **D** Correlation matrix of immune and treatment-related features. **E** Fourfold cross-validation of AUC of prediction probabilities of patients**’** dataset.

On logistic regression multivariate analysis, the six immune cell types and two clinical parameters showed independent predictive value (Fig. 6A). Whereas no prior therapy and increased frequencies of both B-cell subsets were associated with >553.5 IU/mL anti-RBD IgG, prior exposure to anti-CD38 therapy, and the expansion of neutrophils, classical monocytes, and the two T-cell subsets, were associated with lower antibody titers (Fig. 6A, B). The AUC of the logistic regression model was 0.90 (Fig. 6C), with only partial correlation between immune and clinical covariates (Fig. 6D). Any attempt to simplify the model by removing one or more of the parameters described above, significantly impaired its accuracy (data not shown). Cross-validation scores for four folds of the data generated AUC of 0.89, 0.80, 0.88, and 0.95, suggesting that the model may yield accurate predictions to new unseen data (Fig. 6E). Thus, these results identify six immune biomarkers predictive of weak immunogenicity, which are independent of patient’ demographics and clinical characteristics.

## Discussion

The dismal prognosis of COVID-19 in patients with hematological malignancies is well established [38]. There is emerging evidence, suggesting reduced SARS-CoV-2 vaccine effectiveness in these patients [18, 19, 28–30, 39, 20–27]. However, to our knowledge, there is no data on the immune landscape of hematological patients prior vaccination, nor if it can help decipher the mechanism underlying the lack of response (particularly in unexpected cases) and, ultimately, predict seroconversion. Here we performed a comprehensive immunological characterization of hematological patients prior to vaccination, which shows marked differences when compared to HCP of similar age. These analyses also exposed the extent of immunosuppression induced by anticancer treatment and uncovered that immune dysregulation is present before and persists after therapy. Our results translated into cutoffs for broad use of new immune biomarkers to predict antibody response after vaccine for COVID-19 in hematological patients.

As expected, most of the significant differences in the immune landscape of this cohort of patients were observed in the B-cell compartment. More importantly, the marked reduction in the frequency of numerous B-cell subsets observed in patients with B-cell lymphoproliferative disorders, monoclonal gammopathies, and to less extent, Hodgkin lymphoma, translated into lower anti-RBD and anti-S immunoglobulin levels after second-dose SARS-CoV-2 vaccination. Furthermore, due to the multiplex nature of the serological flow cytometry assay used in this study, we unveiled that hematological patients had reduced indexes of both IgG and IgA antibodies. Although the roles of IgA and mucosal immunity in COVID-19 protection remain unknown [40], IgA antibodies are generally considered as the most important immunoglobulin to neutralize infectious pathogens in the respiratory tract.

Seropositivity remains an imperfect proxy for clinically protective immunity against SARS-CoV-2 [41]. Here we reported rates of seroconversion and IgG levels against the RBD fragment of the Spike glycoprotein, which is the major target of neutralizing antibodies. We established a cutoff of 553.5 IU/mL, because it was the median value found in patients and it clearly segregated a few HCP with antibody titers below the cutoff from the remaining control group. Of note, only four HCP developed (mild) COVID-19 after vaccination, which reinforces that the levels of antibody production above this concentration are clinically meaningful to prevent symptomatic infection. Although it was outside the scope of our study, the simultaneous analysis of seroconversion rates and antibody concentration yielded interesting observations. First, vaccination with mRNA-1273 yielded significantly higher anti-RBD IgG titers in hematological patients than BNT162b2, as described elsewhere [42]. Second, although patients with Hodgkin lymphoma may generally produce antibodies, their titers were lower than those of HCP. Third, seroconversion rates between patients with a mature B-cell and PC neoplasm were not considerably different, but immunogenicity was significantly weaker in those with monoclonal gammopathies. Fourth, although seroconversion rates were similar between patients that never received anticancer therapy and those that were off-treatment, antibody production was impaired in the latter. Thus, ongoing and future analysis should focus on antibody concentration beyond seroconversion rates and use if possible, assays that are calibrated against the WHO Standard for cross-study comparison.

Using anti-RBD and anti-S immunoglobulin assays as a surrogate for COVID-19 immunity in lieu of neutralizing antibodies against SARS-CoV-2 is controversial. However, it is reasonable to consider that anti-RBD and anti-S titers would be highly correlated with neutralizing antibody activity [15], and such a correlation was recently shown in a cohort of hematological patients [39]. Another limitation of this study is that antibody titers do not fully account for protection against COVID-19 compared to other forms of immunity such as SARS-CoV-2-specific memory T cells, which may be protective even in seronegative patients. Noteworthy, a reduced T-cell response compared to healthy individuals and patients with solid cancer, has been detected in those with hematological malignancies after SARS-CoV-2 vaccination [43, 44]. Although measuring T-cell specificity was outside the scope of this study, we showed that, when compared to HCP of similar age, the T-cell compartment was significantly altered in hematological patients, particularly those with a monoclonal gammopathy, and in those on or off-treatment. Interestingly, we observed in these patients a pronounced reduction of Tfh cell types, which play an essential role in regulating the germinal center reaction and, consequently, the generation of high-affinity antibodies [45, 46]. Thus, our results may shed some light on the low rate of T-cell responses in hematological patients [39, 44], particularly in seronegative cases [44].

In MM, a positive correlation between serological response and CD19+ or CD4+ lymphocyte counts was most recently observed, while CD8+ T-cell counts were negatively correlated [47]. In another study that included a more heterogeneous population of patients with hematological malignancies, the strong correlation between CD19+ B cells and serological response was confirmed, and there was no correlation between antibody concentration and CD4+ or CD8+ T cells [39]. Of note, CD19+ B-cell counts had independent predictive value in the MM cohort and in the larger series of patients with hematological malignancies [39, 47]. Here we uncovered the presence of a distinct immunotype that, although significantly associated with some disease and treatment-related features, identified patients with a B-cell and a PC neoplasm with more frequent seronegativity and lower antibody titers after second-dose vaccination. More importantly, our results show that CD4+, CD8+, and CD19+ lymphocytes, and many of their respective subsets are essential for vaccine effectiveness and should be monitored to predict immunogenicity. Increased numbers of neutrophils and classical monocytes were significantly associated with poor antibody response, and this could be related to their immunosuppressive phenotype [48].

The influence of anticancer treatment and timing of the immune response to SARS-CoV-2 remains a topic of clinical interest and controversy. Thus, and until more definitive data emerges, decisions around delivery or interruption of anticancer therapy should be based on individual risk-benefit assessment incorporating factors including cancer prognosis and patient comorbidities. Here we aimed to provide new biomarkers to help on such decisions and thus, we identified six immune cell types that depict patients’ immune landscape (i.e., percentages of immunosuppressive cells and of T- and B-cell subsets representative of the cellular and humoral immunity) and are independent of other disease features in order to predict immunogenicity prior to vaccination. Despite the complex phenotype of some immune cell types, which is the result of the holistic and computational flow cytometry analysis we performed, there are standardized methods for their quantification [49]. Immune profiling prior vaccination for COVID-19 could be particularly informative in patients that are off active treatment, who are expected to become seropositive but may have poor antibody response due to tumor-related immune dysregulation and may benefit from third-dose vaccination.

Interestingly, we found that hematological patients with undocumented SARS-CoV-2 infection developed median antibody titers after two doses that were similar to those found in HCP. Thus, our data reproduces previous findings in non-cancer patients [50, 51] and could suggest that a third challenge to SARS-CoV-2 antigens can improve immunogenicity. Just recently, the US Food and Drugs Administration amended the emergency use authorization for the BNT162b2 and mRNA-1273 COVID-19 vaccines to allow for an additional dose for certain people with compromised immune systems. That group includes, specifically, individuals over 65 years old, solid organ transplant recipients, or those who are diagnosed with conditions that are considered to be equally immunocompromised. Our study exposes the depth of immune dysregulation in patients with hematological malignancies and urges reflection on whether immune profiling before boosting is warranted to identify optimal timing of third-dose vaccination.

## Supplementary information

Supplemental Material

Checklist_21-BCJ-0653
