# Supplementary material for: Immune biomarkers to predict SARS-CoV-2 vaccine effectiveness in patients with hematological malignancies
Source: Blood Cancer J. 2021 Dec 14;11(12):202. doi: 10.1038/s41408-021-00594-1 (PMC8669666; doi:10.1038/s41408-021-00594-1)

**TABLE OF CONTENTS**

Supplemental Table 1………………………………………………………………………….2

Supplemental Table 2………………………………………………………………………….3

Supplemental Table 3………………………………………………………………………….7

Supplemental Figure 1…………………………………………………………………………9

Supplemental Figure 2…………..………….………………………………………………..12

Supplemental Figure 3…………..………….………………………………………………..15

Supplemental Figure 4…………..………….………………………………………………..18

Supplemental Figure 5…………..………….………………………………………………..19

Supplemental Figure 6…………..………….………………………………………………..20

Supplemental Figure 7……………………………………………………………………….21

**Supplemental Table 1.** Description of the antibodies used in this study.

| **Panel** | **Antigen** | **Label** | **Clone** | **Cat #** | **Company** |
| --- | --- | --- | --- | --- | --- |
| Granulocytes and APC | CD36 | FITC | CLB-IVC7 | IM1613 | Sanquin |
|  | SLAN | PE | DD-1 | 130-093-029 | Miltenyi Biotec |
|  | CD33 | PerCP-Cy5.5 | P67.6 | 333146 | BDB |
|  | CD16 | PE-Cy7 | 3G8 | 560716 | BDB |
|  | CD123 | APC | AC145 | 130-090-901 | Miltenyi Biotec |
|  | CD14 | APCH7 | MϕP9 | 641394 | BDB |
|  | HLA-DR | PacB | L243 | 307633 | Biolegend |
|  | CD45 | OC515 | GA90 | CYT-450C | Cytognos |
| T cells | CD45RA | FITC |  | 335039 | BDB |
|  | CD25 | PE | 2A3 | 341011 | BDB |
|  | CD8 | PerCP-Cy5.5 | SK1 | 341050 | BDB |
|  | CD279 | PE-Cy7 | PD1.3 | A78885 | Beckman Coulter |
|  | CXCR5 | APC |  | FAB190A | R&D |
|  | CD197 | APC-Cy7 |  | 353212 | Biolegend |
|  | CD4 | PacB | 13B8.2 | B49197 | Beckman Coulter |
|  | CD127 | BV510 | HIL-7R-M21 | 563086 | BDB |
| B cells | IgE+IgA | FITC |  | H15701 / 130-093-071 | Life Technologies / Miltenyi Biotec |
|  | IgG+IgA | PE | G18-145 | 555787 / 130-093-128 | BD / Miltenyi Biotec |
|  | IgD | PerCP-Cy5.5 | IA6-2 | 348208 | Biolegend |
|  | CD19 | PE-Cy7 | J3-119 | IM3628 | Beckman Coulter |
|  | CD21 | APC | B-ly4 | 559867 | BD |
|  | CD38 | APCH7 | HB7 | 656646 | BD |
|  | CD27 | BV421 | M-T271 | 562513 | BD |
|  | IgM | BV510 | MHM-88 | 314521 | Biolegend |

BDB, Beckton Dickinson Biosciences

**Supplemental Table 2.** Immunophenotypic profile of the 59 immune cell-types identified by automated clustering using *FlowCT*.

| **Immune subset** | **Immune cell type** | **Immunophenotype** |
| --- | --- | --- |
| Granulocytes | Basophils | SSC^lo^ FSC^hi^ CD45^lo^ CD33^+^ CD16^-^ HLA-DR^-^ CD123^+^ |
|  | Eosinophils | SSC^hi^ FSC^hi^ CD45^hi^ CD33^+^ CD16^-^ HLA-DR^-^ CD123^-^ |
|  | Neutrophils | SSC^hi^ FSC^hi^ CD45^+^ CD33^het^ CD16^hi^ HLA-DR^-^ CD123^-^ |
| APC | Classical monocytes | SSC^lo^ FSC^lo^ CD45^hi^ CD33^+^ CD16^-^ CD14^+^ CD36^+^ HLA-DR^+^ CD123^-^ SLAN^-^ |
|  | Intermediate monocytes | SSC^lo^ FSC^lo^ CD45^hi^ CD33^+^ CD16^+^ CD14^+^ CD36^+^ HLA-DR^hi^ CD123^-^ SLAN^-^ |
|  | SLAN^-^ Non-Classical monocytes | SSC^lo^ FSC^lo^ CD45^hi^ CD33^+^ CD16^+^ CD14^-^ CD36^+^ HLA-DR^hi^ CD123^-^ SLAN^-^ |
|  | SLAN^+^ Non-Classical monocytes | SSC^lo^ FSC^lo^ CD45^high^ CD33^+^ CD16^+^ CD14^-^ CD36^+^ HLA-DR^hi^ CD123^-^ SLAN^+^ |
|  | Myeloid dendritic cells (mDC) | SSC^lo^ FSC^lo^ CD45^hi^ CD33^+^ CD16^-^ CD14^-^ CD36^lo^ HLA-DR^hi^ CD123^-^ SLAN^-^ |
|  | Plasmacytoid dendritic cells (pDC) | SSC^lo^ FSC^lo^ CD45^hi^ CD33^lo^ CD16^-^ CD14^-^ CD36^lo^ HLA-DR^+^ CD123^+^ SLAN^-^ |
| T cells |  |  |
|  | CD4^+^ Naive | CD4^+^ CD8^-^ CD197^+^ CD45RA^+^ CD127^+^ CD25^het^ PD1^-^ CXCR5^-^ |
|  | CD4^+^ CM ^neg^ | CD4^+^ CD8^-^ CD197^+^ CD45RA^-^ CD127^-^ CD25^-^ PD1^-^ CXCR5^-^ |
|  | CD4^+^ CM CD127^+^ | CD4^+^ CD8^-^ CD197^+^ CD45RA^-^ CD127^+^ CD25^-^ PD^1+^ CXCR5^-^ |
|  | CD4^+^ CM CD127^+^ CD25^+^ | CD4^+^ CD8^-^ CD197^+^ CD45RA^-^ CD127^+^ CD25^+^ PD1^+^ CXCR5^-^ |
|  | CD4^+^ CM CD127^lo^ PD1^+^ | CD4^+^ CD8^-^ CD197^+^ CD45RA^-^ CD127^lo^ CD25^-^ PD1^+^ CXCR5^-^ |
|  | CD4^+^ CM CD127^+^ PD1^+^ | CD4^+^ CD8^-^ CD197^+^ CD45RA^-^ CD127^+^ CD25^-^ PD1^+^ CXCR5^-^ |
|  | CD4^+^ EM CD127^lo^ | CD4^+^ CD8^-^ CD197^-^ CD45RA^+^ CD127^lo^ CD25^-^ PD1^-^ CXCR5^-^ |
|  | CD4^+^ EM CD127^+^ | CD4^+^ CD8^-^ CD197^-^ CD45RA^-^ CD127^+^ CD25^-^ PD1^-^ CXCR5^-^ |
|  | CD4^+^ EM CD127^+^ CD25^+^ | CD4^+^ CD8^-^ CD197^-^ CD45RA^-^ CD127^+^ CD25^+^ PD1^-^ CXCR5^-^ |
|  | CD4^+^ EM CD127^lo^ PD1^+^ | CD4^+^ CD8^-^ CD197^-^ CD45RA^-^ CD127^lo^ CD25^-^ PD1^+^ CXCR5^-^ |
|  | CD4^+^ EM CD127^+^ PD1^+^ | CD4^+^ CD8^-^ CD197^-^ CD45RA^-^ CD127^+^ CD25^-^ PD1^+^ CXCR5^-^ |
|  | CD4^+^ EM CD127^+^ CD25^+^ PD1^+^ | CD4^+^ CD8^-^ CD197^-^ CD45RA^-^ CD127^+^ CD25^+^ PD1^+^ CXCR5^-^ |
|  | CD4^+^ TEMRA CD127^lo^ | CD4^+^ CD8^-^ CD197^-^ CD45RA^+^ CD127^lo^ CD25- PD1^-^ CXCR5^-^ |
|  | CD4^+^ TEMRA CD127^+^ | CD4^+^ CD8^-^ CD197^-^ CD45RA^+^ CD127^+^ CD25^-^ PD1^-^ CXCR5^-^ |
|  | Naïve Treg | CD4^+^ CD8^-^ CD197^+^ CD45RA^+^ CD127^lo^ CD25^+^ PD1^-^ CXCR5^-^ |
|  | CM Treg | CD4^+^ CD8^-^ CD197^+^ CD45RA^-^ CD127^lo^ CD25^+^ PD1^-^ CXCR5^-^ |
|  | EM Treg | CD4^+^ CD8^-^ CD197^-^ CD45RA^-^ CD127^lo^ CD25^+^ PD1^-^ CXCR5^-^ |
|  | Tfh | CD4^+^ CD8^-^ CD197^+^ CD45RA^-^ CD127^+^ CD25^-^ PD1^+^ CXCR5^+^ |
|  | Tfh-like CM | CD4^+^ CD8^-^ CD197^+^ CD45RA^-^ CD127^+^ CD25^-^ PD1^-^ CXCR5^+^ |
|  | CD8^+^ naïve | CD4^-^ CD8^+^ CD197^+^ CD45RA^+^ CD127^+^ CD25^-^ PD1^-^ CXCR5^-^ |
|  | CD8^+^ CM CD127^lo^ | CD4^-^ CD8^+^ CD197^+^ CD45RA^-^ CD127^lo^ CD25^-^ PD1^-^ CXCR5^-^ |
|  | CD8^+^ CM CD127^+^ | CD4^-^ CD8^+^ CD197^+^ CD45RA^-^ CD127^+^ CD25^-^ PD1^-^ CXCR5^-^ |
|  | CD8^+^ CM CD127^+^ CD25^+^ | CD4^-^ CD8^+^ CD197^+^ CD45RA^-^ CD127^+^ CD25^+^ PD1^-^ CXCR5^-^ |
|  | CD8^+^ EM CD127^lo^ | CD4^-^ CD8^+^ CD197^-^ CD45RA^-^ CD127^lo^ CD25^-^ PD1^-^ CXCR5^-^ |
|  | CD8^+^ EM CD127^+^ | CD4^-^ CD8^+^ CD197^-^ CD45RA^-^ CD127^+^ CD25^-^ PD1^-^ CXCR5^-^ |
|  | CD8^+^ EM PD1^+^ | CD4^-^ CD8^+^ CD197^-^ CD45RA^-^ CD127^-^ CD25^-^ PD1^+^ CXCR5^-^ |
|  | CD8^+^ EM CD127^+^ PD1^+^ | CD4^-^ CD8^+^ CD197^-^ CD45RA^-^ CD127^+^ CD25^-^ PD1^+^ CXCR5^-^ |
|  | CD8^+^ EM CXCR5^+^ CD127^+^ PD1^+^ | CD4^-^ CD8^+^ CD197^-^ CD45RA^-^ CD127^+^ CD25^-^ PD1^+^ CXCR5^+^ |
|  | CD8^+^ TEMRA CD127^lo^ | CD4^-^ CD8^+^ CD197^-^ CD45RA^+^ CD127^lo^ CD25^-^ PD1^-^ CXCR5^-^ |
|  | CD8^+^ TEMRA CD127^+^ | CD4^-^ CD8^+^ CD197^-^ CD45RA^+^ CD127^+^ CD25^-^ PD1^-^ CXCR5^-^ |
|  | CD8^+^ TEMRA CD127^+^ PD1^+^ | CD4^-^ CD8^+^ CD197^-^ CD45RA^+^ CD127^+^ CD25^-^ PD1^+^ CXCR5^-^ |
|  | CD8^+^ TEMRA PD1^+^ | CD4^-^ CD8^+^ CD197^-^ CD45RA^+^ CD127^-^ CD2^5-^ PD1^+^ CXCR5^-^ |
| B cells | Naive CD21^-^ | CD27^-^ CD38^-^ CD21^-^ IgD^+^ IgM^+^ IgA^-^ IgG^-^ IgE^-^ |
|  | Naive CD21^+^ | CD27^-^ CD38^-^ CD21^+^ IgD^+^ IgM^+^ IgA^-^ IgG^-^ IgE^-^ |
|  | Transitional | CD27^-^ CD38^+^ CD21^+^ IgD^-^ IgM^lo^ IgA^-^ IgG^-^ IgE^-^ |
|  | IgM^+^D^+^ CD27^+^ CD21^-^ memory | CD27^+^ CD38^-^ CD21^-^ IgD^+^ IgM^+^ IgA^-^ IgG^-^ IgE^-^ |
|  | IgM^+^D^+^ CD27^+^ CD21^+^ memory | CD27^+^ CD38^-^ CD21^-^ IgD^+^ IgM^+^ IgA^-^ IgG^-^ IgE^-^ |
|  | IgM^+^D^-^ CD27^-^ CD21^-^ memory | CD27^-^ CD38^-^ CD21^-^ IgD^-^ IgM^+^ IgA^-^ IgG^-^ IgE^-^ |
|  | IgM^+^D^-^ CD27^-^ CD21^+^ memory | CD27^-^ CD38^-^ CD21^+^ IgD^-^ IgM^+^ IgA^-^ IgG^-^ IgE^-^ |
|  | IgM^+^D^-^ CD27^+^ CD21^-^ memory | CD27^+^ CD38^-^ CD21^-^ IgD^-^ IgM^+^ IgA^-^ IgG^-^ IgE^-^ |
|  | IgM^+^D^-^ CD27^+^ CD21^+^ memory | CD27^+^ CD38^-^ CD21^+^ IgD^-^ IgM^+^ IgA^-^ IgG^-^ IgE^-^ |
|  | IgG^+^ CD27^-^ CD21^-^ memory | CD27^-^ CD38^-^ CD21^-^ IgD^-^ IgM^-^ IgA^-^ IgG^+^ IgE^-^ |
|  | IgG^+^ CD27^-^ CD21^+^ memory | CD27^-^ CD38^-^ CD21^+^ IgD^-^ IgM^-^ IgA^-^ IgG^+^ IgE^-^ |
|  | IgG^+^ CD27^+^ CD21^lo^ memory | CD27^+^ CD38^-^ CD21^lo^ IgD^-^ IgM^-^ IgA^-^ IgG^+^ IgE^-^ |
|  | IgG^+^ CD27^+^ CD21^+^ memory | CD27^+^ CD38^-^ CD21^+^ IgD^-^ IgM^-^ IgA^-^ IgG^+^ IgE^-^ |
|  | IgA^+^ CD27^-^ CD21^-^ memory | CD27^-^ CD38^-^ CD21^-^ IgD- IgM^-^ IgA^+^ IgG^-^ IgE^-^ |
|  | IgA^+^ CD27^-^ CD21^+^ memory | CD27^-^ CD38^-^ CD21^+^ IgD^-^ IgM^-^ IgA^+^ IgG^-^ IgE^-^ |
|  | IgA^+^ CD27^+^ CD21^+^ memory | CD27^+^ CD38^-^ CD21^+^ IgD^-^ IgM^-^ IgA^+^ IgG^-^ IgE^-^ |
|  | IgG^+^IgM^+^ circulating PC | CD27^hi^ CD38^hi^ CD21^-^ IgD^-^ IgM^+^ IgA^-^ IgG^+^ IgE^-^ |
|  | IgA circulating PC | CD27^hi^ CD38^hi^ CD21^-^ IgD^-^ IgM^-^ IgA^+^ IgG^-^ IgE^-^ |

APC, antigen-presenting cells; CM, central memory; EM, effector memory; TEMRA, effector memory T-cell re-expressing CD45RA; Tfh, follicular helper T cell, Treg, regulatory T cell; Ig, immunoglobulin; PC, plasma cells.

**Supplemental Table 3.** Optimal cutoffs in percentages (%) and absolute counts (cells/uL) for each immune cell-type.

| Immune subset | Cutoff (%) | Cutoff  (Cells/uL) | AUC | *P value* |
| --- | --- | --- | --- | --- |
| Basophils | 1.12 | 66 | 0.571 | 0.193 |
| Eosinophils | 3.09 | 180 | 0.5 | 0.442 |
| Neutrophils | 60 | 3550 | 0.58 | 0.077 |
| Classical monocytes | 10 | 631 | 0.59 | 0.019 |
| Intermediate monocytes | 0.67 | 39 | 0.57 | 0.051 |
| SLAN- non-classical monocytes | 0.37 | 22 | 0.58 | 0.019 |
| SLAN+ non-classical monocytes | 0.01 | 1 | 0.6 | 0.100 |
| Myeloid dendritic cells | 0.37 | 22 | 0.611 | 0.050 |
| Plasmacytoid dendritic cells | 0.14 | 8 | 0.7 | 0.003 |
| CD4+ CM CD127low PD1+ | 0.03 | 2 | 0.61 | 0.006 |
| CD4+ CM CD127+ | 1.61 | 94 | 0.73 | 0.001 |
| CD4+ CM CD127+ CD25+ | 0.34 | 20 | 0.67 | 0.001 |
| CD4+ CM CD127+ PD1+ | 0.15 | 9 | 0.63 | 0.011 |
| CD4+ CM neg | 0.16 | 9 | 0.65 | 0.005 |
| CD4+ EM CD127low | 0.21 | 12 | 0.64 | 0.001 |
| CD4+ EM CD127low PD1+ | 0.49 | 28 | 0.58 | 0.021 |
| CD4+ EM CD127+ | 1.07 | 62 | 0.53 | 0.511 |
| CD4+ EM CD127+ CD25+ | 0.65 | 38 | 0.66 | 0.004 |
| CD4+ EM CD127+ CD25+ PD1+ | 0.29 | 17 | 0.67 | 0.007 |
| CD4+ EM CD127+ PD1+ | 0.47 | 28 | 0.59 | 0.145 |
| CD4+ Naive | 1.35 | 79 | 0.75 | 0.000 |
| CD4+ TEMRA CD127low | 0.11 | 6 | 0.51 | 0.224 |
| CD4+ TEMRA CD127+ | 0.01 | 1 | 0.51 | 0.215 |
| Tfh | 0.17 | 10 | 0.59 | 0.042 |
| Tfh-like CM | 0.50 | 29 | 0.73 | 0.000 |
| Treg CM | 0.17 | 10 | 0.65 | 0.004 |
| Treg EM | 0.74 | 43 | 0.48 | 0.515 |
| Treg Naïve | 0.05 | 3 | 0.73 | 0.000 |
| CD8+ CM CD127low | 0.34 | 20 | 0.52 | 0.326 |
| CD8+ CM CD127+ | 0.49 | 28 | 0.66 | 0.008 |
| CD8+ CM CD127+ CD25+ | 0.07 | 4 | 0.56 | 0.018 |
| CD8+ EM CD127low | 0.84 | 49 | 0.67 | 0.003 |
| CD8+ EM CD127+ | 1.05 | 61 | 0.54 | 0.303 |
| CD8+ EM CD127+ PD1+ | 0.16 | 9 | 0.66 | 0.001 |
| CD8+ EM CXCR5+ CD127+ PD1+ | 0.06 | 3 | 0.52 | 0.444 |
| CD8+ EM PD1+ | 0.42 | 25 | 0.53 | 0.154 |
| CD8+ Naive | 1.15 | 67 | 0.71 | 0.001 |
| CD8+ TEMRA CD127low | 0.99 | 58 | 0.56 | 0.227 |
| CD8+ TEMRA CD127+ | 0.36 | 21 | 0.44 | 0.589 |
| CD8+ TEMRA CD127+ PD1+ | 0.03 | 2 | 0.69 | 0.002 |
| CD8+ TEMRA PD1+ | 0.08 | 5 | 0.52 | 0.124 |
| Naïve CD21- | 0.01 | 1 | 0.71 | <0.001 |
| Naïve CD21+ | 0.47 | 27 | 0.75 | <0.001 |
| Transitional | 0.02 | 1 | 0.65 | 0.001 |
| IgM+ IgD+ memory CD27+ CD21- | 0.01 | 0.3 | 0.79 | <0.001 |
| IgM+ IgD+ memory CD27+ CD21+ | 0.02 | 1 | 0.8 | <0.001 |
| IgM+ memory CD27- CD21- | 0.002 | 0.1 | 0.74 | <0.001 |
| IgM+ memory CD27- CD21+ | 0.002 | 0.1 | 0.76 | <0.001 |
| IgM+ memory CD27+ CD21- | 0.001 | 0.03 | 0.8 | <0.001 |
| IgM+ memory CD27+ CD21+ | 0.01 | 1 | 0.81 | <0.001 |
| IgG+ memory CD27- CD21- | 0.003 | 0.2 | 0.68 | <0.001 |
| IgG+ memory CD27- CD21+ | 0.01 | 0.3 | 0.77 | <0.001 |
| IgG+ memory CD27+ CD21low | 0.01 | 0.5 | 0.8 | <0.001 |
| IgG+ memory CD27+ CD21+ | 0.02 | 1 | 0.78 | <0.001 |
| IgA+ memory CD27- CD21- | 0.001 | 0.1 | 0.73 | <0.001 |
| IgA+ memory CD27- CD21+ | 0.003 | 0.2 | 0.77 | <0.001 |
| IgA+ memory CD27+ CD21+ | 0.02 | 1 | 0.81 | <0.001 |
| IgG+ IgM+ circulanting PCs | 0.0004 | 0.02 | 0.65 | <0.001 |
| IgA+ circulating PCs | 0.001 | 0.1 | 0.69 | 0.001 |

APC, antigen-presenting cells; AUC, area under the receiver operating characteristics curve; CM, central memory; EM, effector memory; TEMRA, effector memory T cell re-expressing CD45RA; Tfh, follicular helper T cell, Treg, regulatory T cell; Ig, immunoglobulin; PC, plasma cells.

**Supplemental Figure 1.** Gating strategy of immune cell subsets identified by *FlowCT*. **(A)** Uniform manifold approximation and projection (UMAP) and dot plots showing the distribution of several myeloid subsets: eosinophils, neutrophils, basophils, antigen presenting cells (APC) and lymphocytes, considering their respective size (FSC) and complexity (SSC) as well as expression of CD45, HLA-DR, CD123, CD33 and CD16 biomarkers. **(B)** UMAP and dot plots displaying the distribution of APC subsets according to the expression levels of CD14, CD16, CD45, CD36, CD123, HLA-DR and SLAN: classical, intermediate, SLAN- and SLAN+ non-classical monocytes, myeloid and plasmacytoid dendritic cells (mDC and pDC, respectively). **(C)** UMAP and dot plots showing several subpopulations within CD19^+^ compartment, considering the expression of CD27, CD38, CD21 and immunoglobulins D, M, A and G (IgD, IgM, IgA and IgG, respectively). **(D)** UMAP and dot plots displaying T-cell subsets according to the expression of CD4, CD8, CD127, CD25, CD197, CD45RA, CD279 and CXCR5 within both CD4 and CD8 compartments. CM, central memory; EM, effector memory; TEMRA, effector memory re-expressing CD45RA; Tfh, helper folicular T cells; Treg; regulatory T cells.


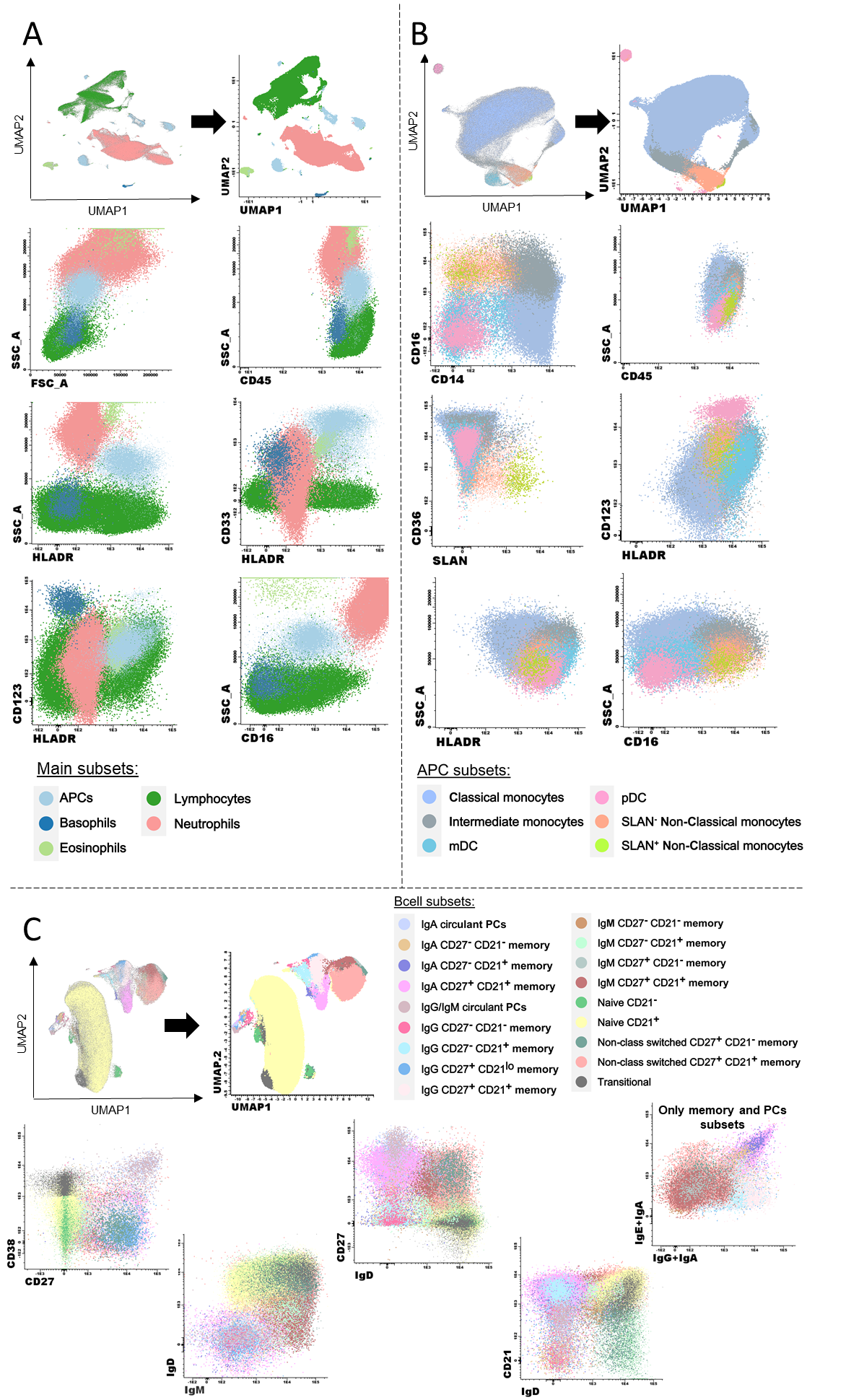


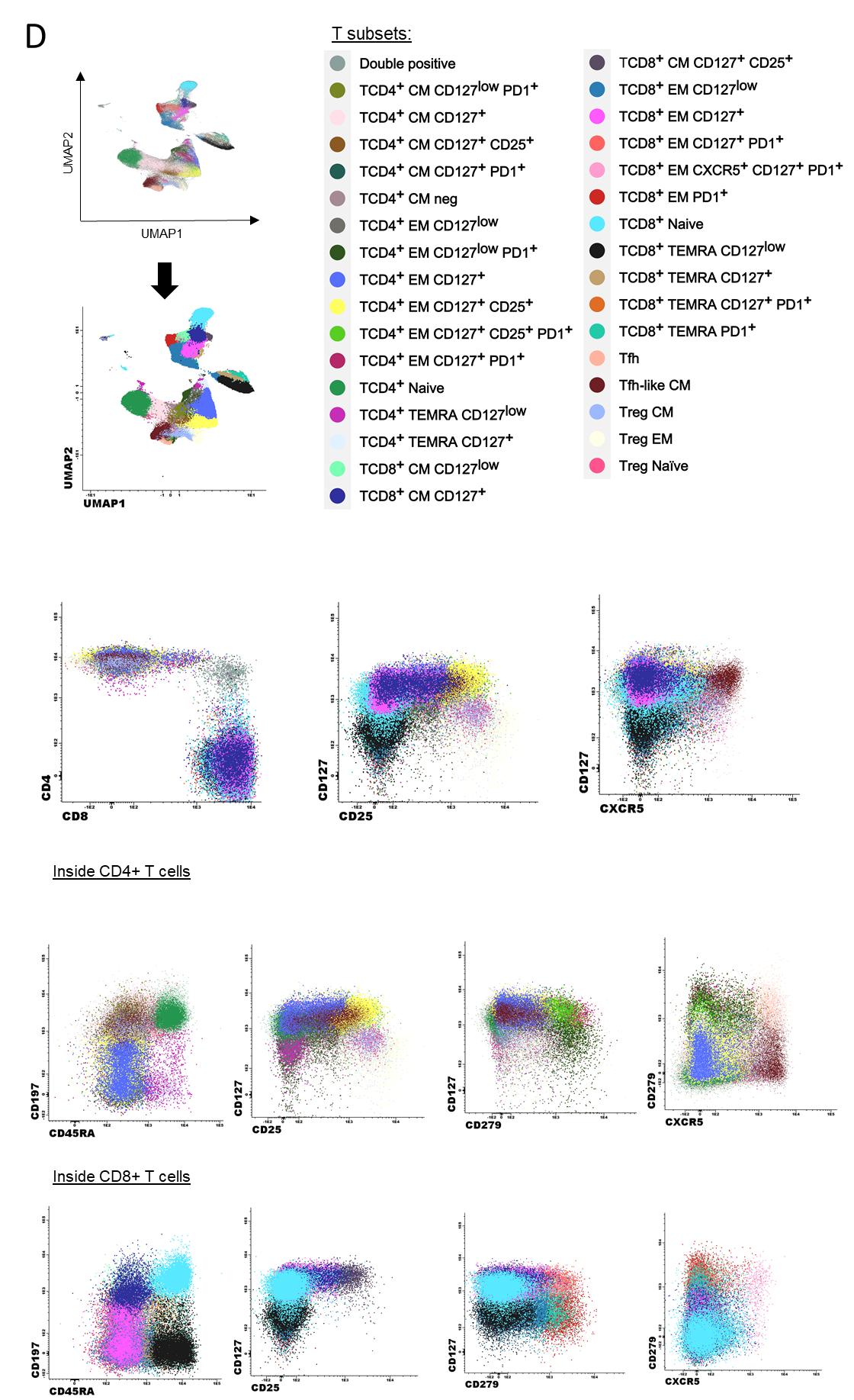


**Supplemental Figure 2.** Relative distribution of the 59 immune cell-types identified in health care practitioners (HCP) and patients with a B-cell neoplasm, Hodgkin lymphoma (HL) and monoclonal gammopathy (MG). Percentages of **(A)** granulocytes, **(B)** antigen-presenting cells, **(C)** CD4+ and **(D)** CD8+ T cells, as well as **(E)** B cells. Statistical significance among all comparisons is detailed in Figure 2.

**A)** Granulocytes


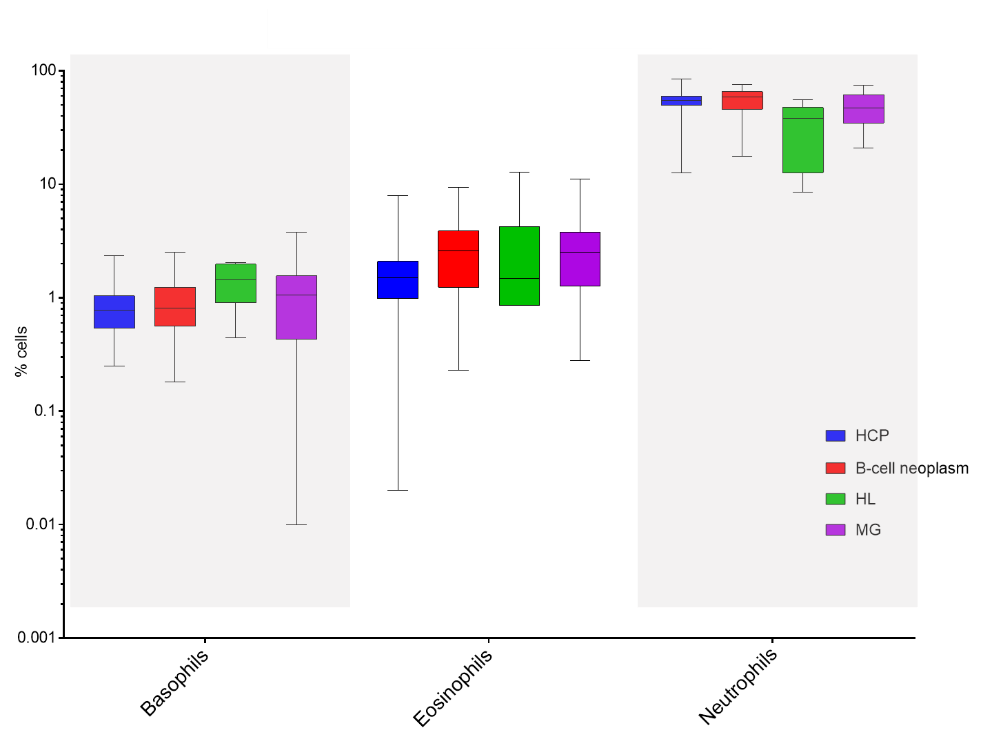


**B)** Antigen-presenting cells


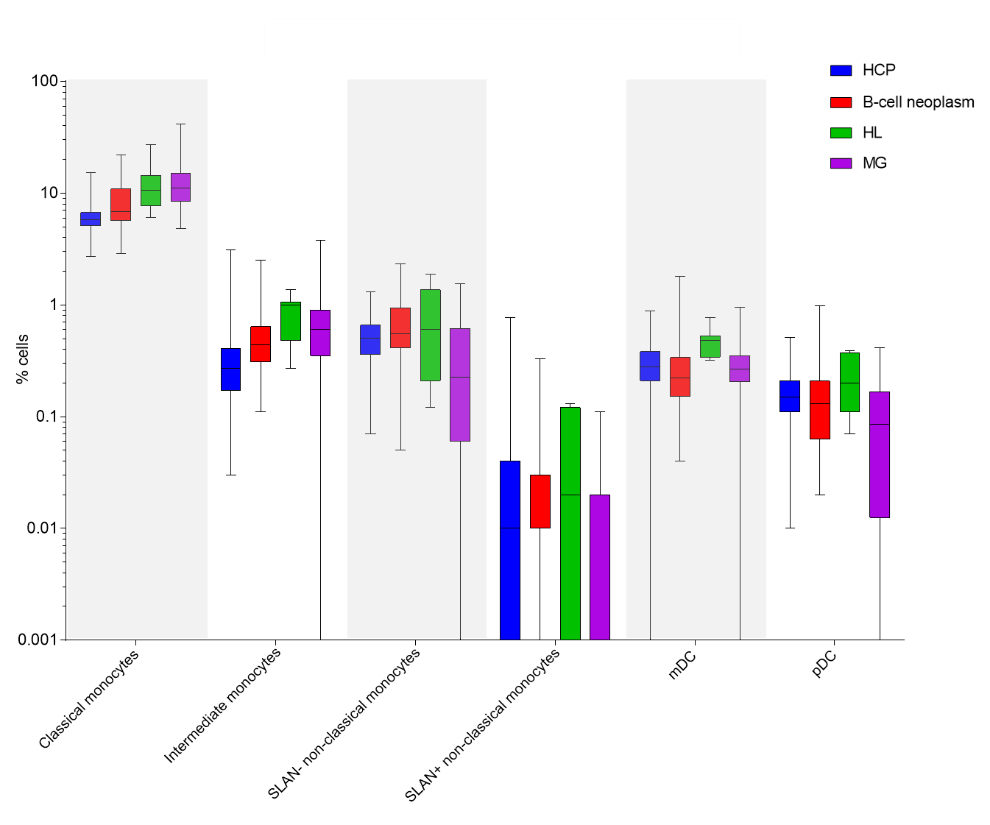


**C)** CD4 T cells


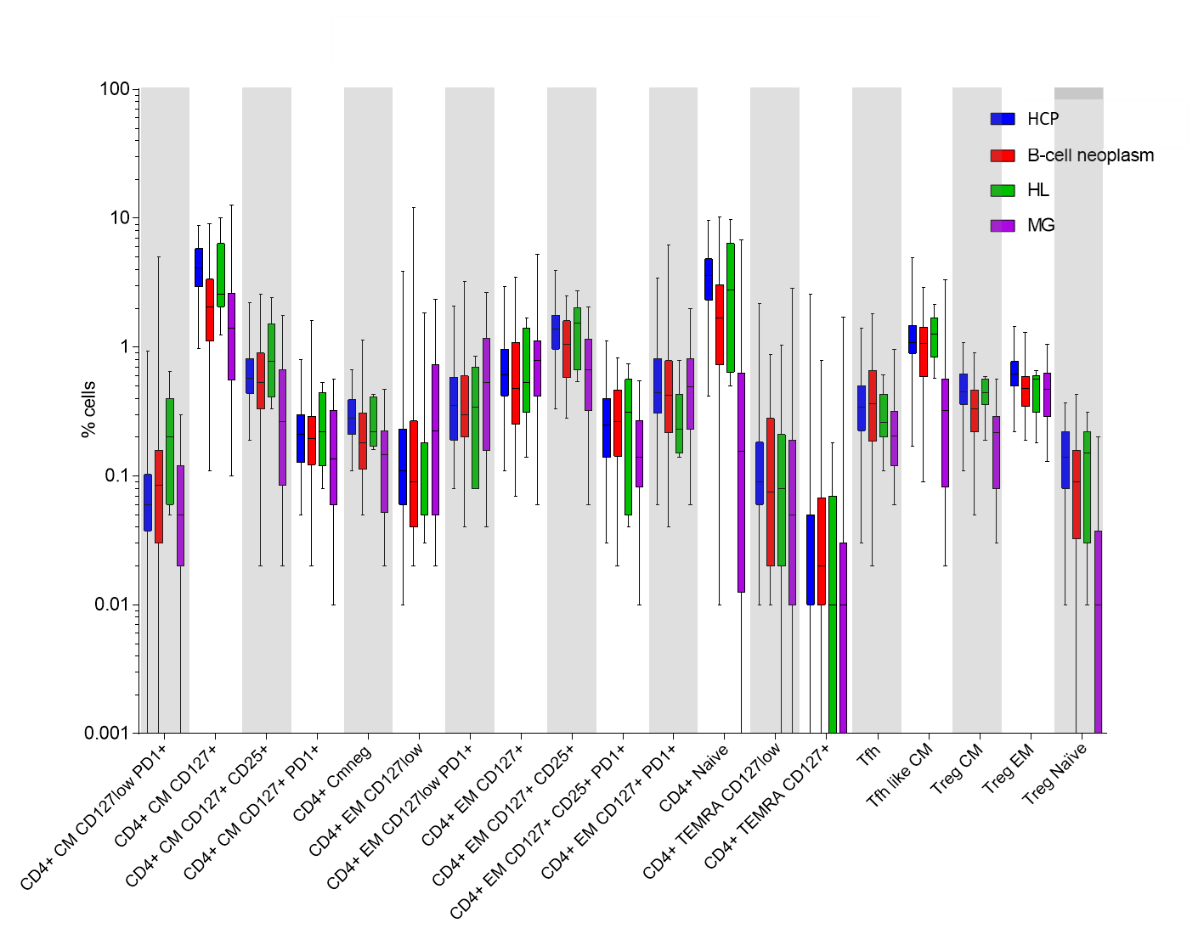


**D)** CD8 T cells


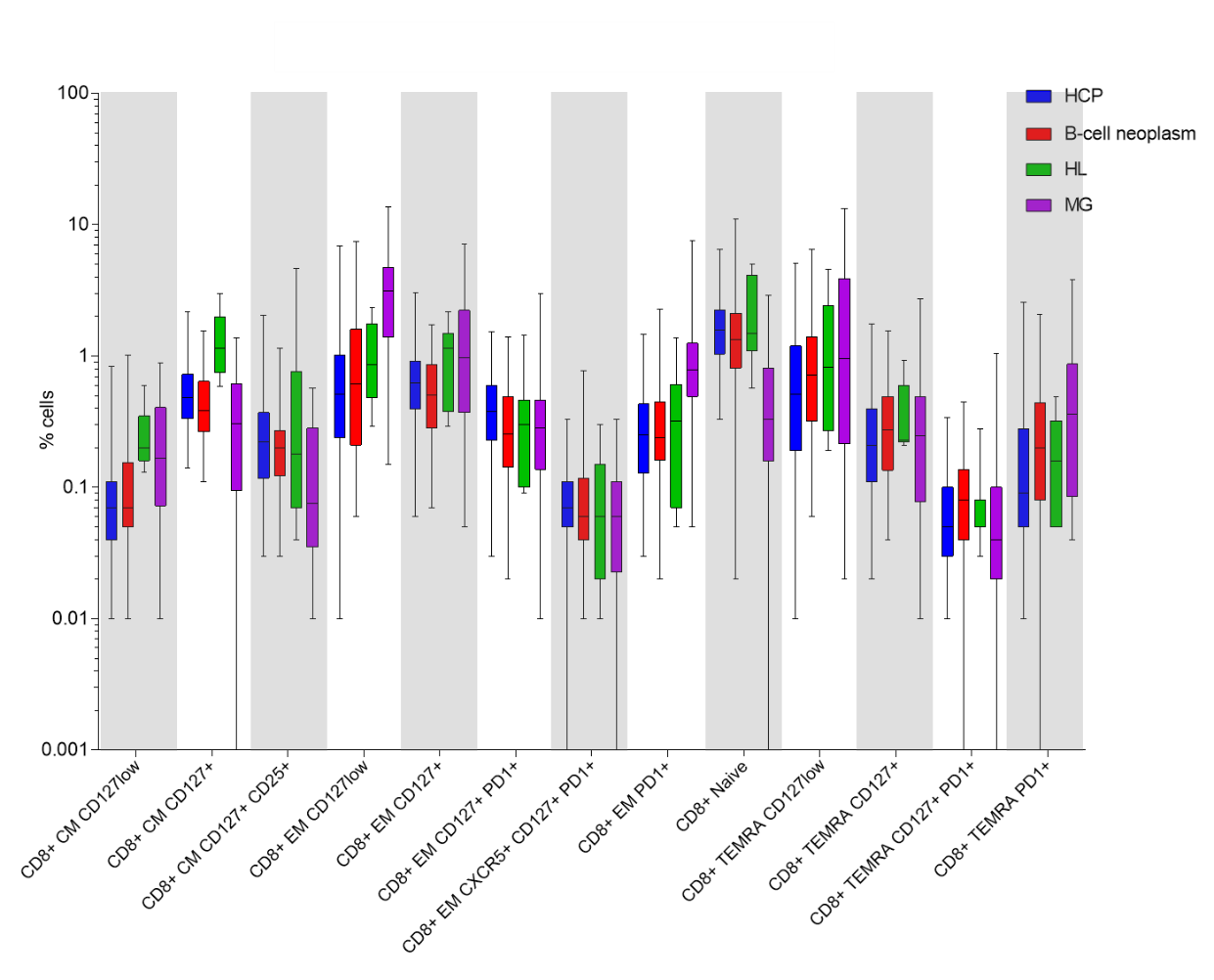


**E)** B cells


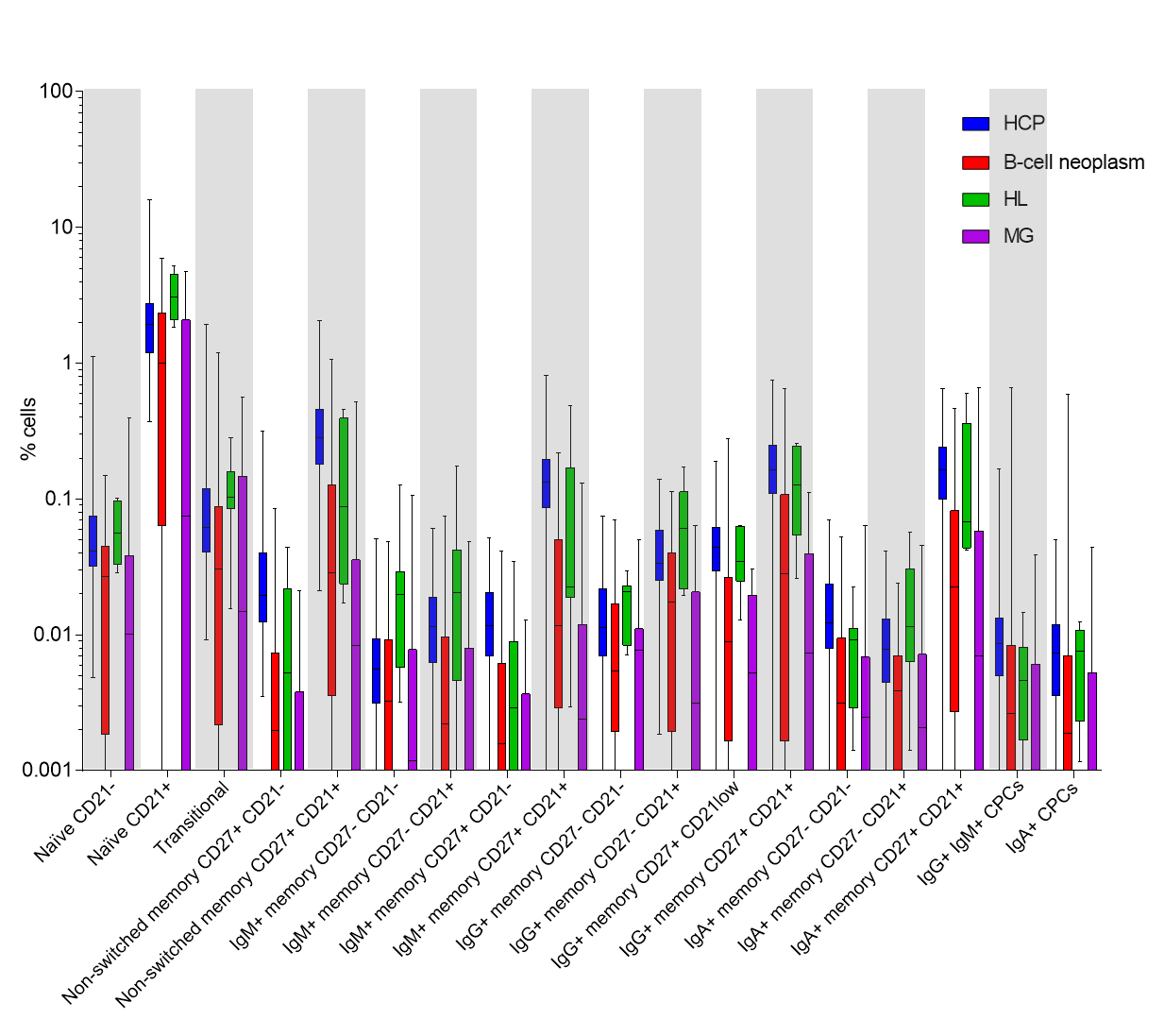


**Supplemental Figure 3.** Relative distribution of the 59 immune cell-types identified in health care practitioners (HCP), hematological patients that had never received treatment (No tx), those that were on (Active tx,) and patients that were off-treatment (Post-tx,) before vaccination. Percentages of **(A)** granulocytes, **(B)** antigen-presenting cells, **(C)** CD4+ and **(D)** CD8+ T cells, as well as **(E)** B cells. Statistical significance among all comparisons is detailed in Figure 3.

**A)** Granulocytes


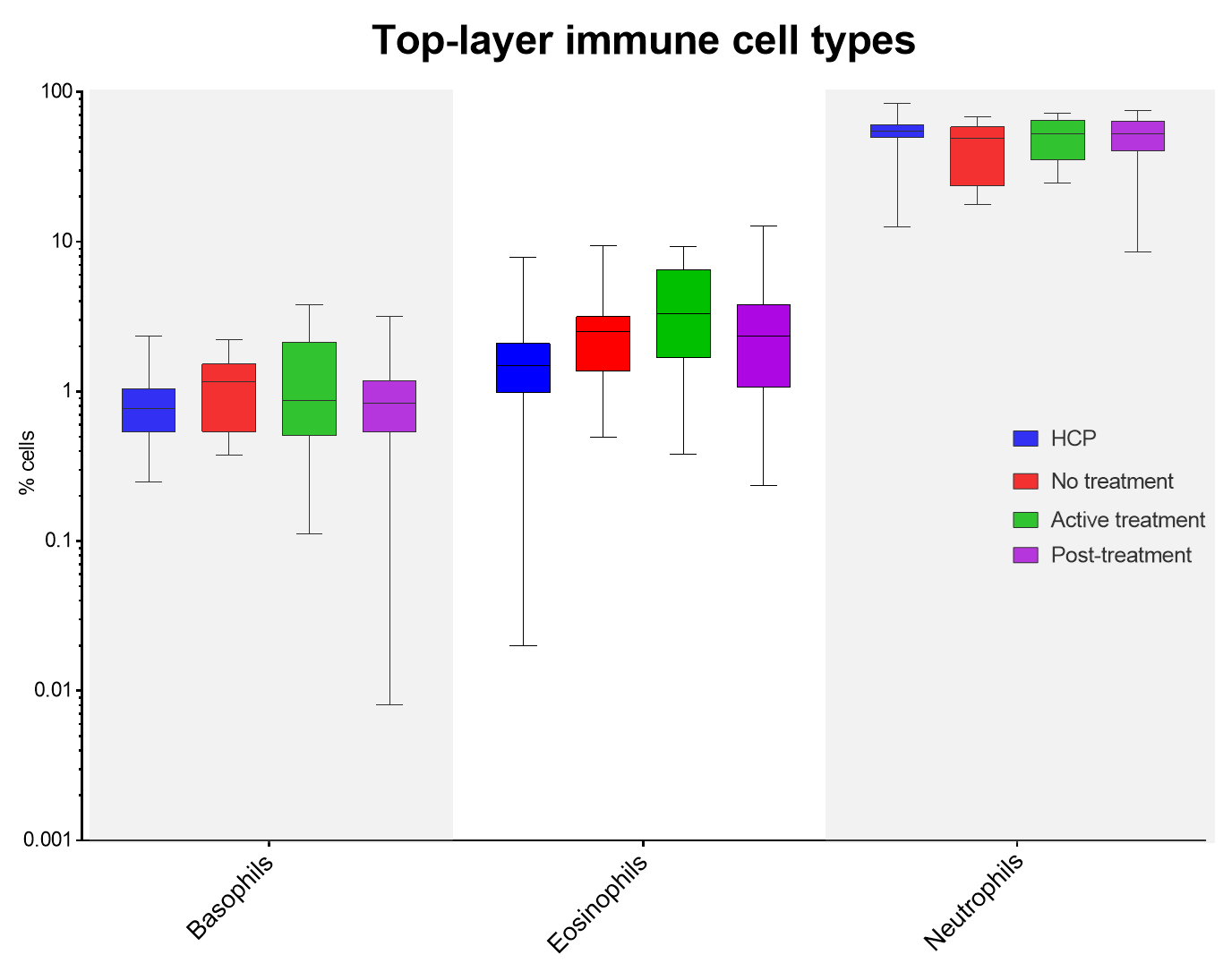


**B)** Antigen-presenting cells


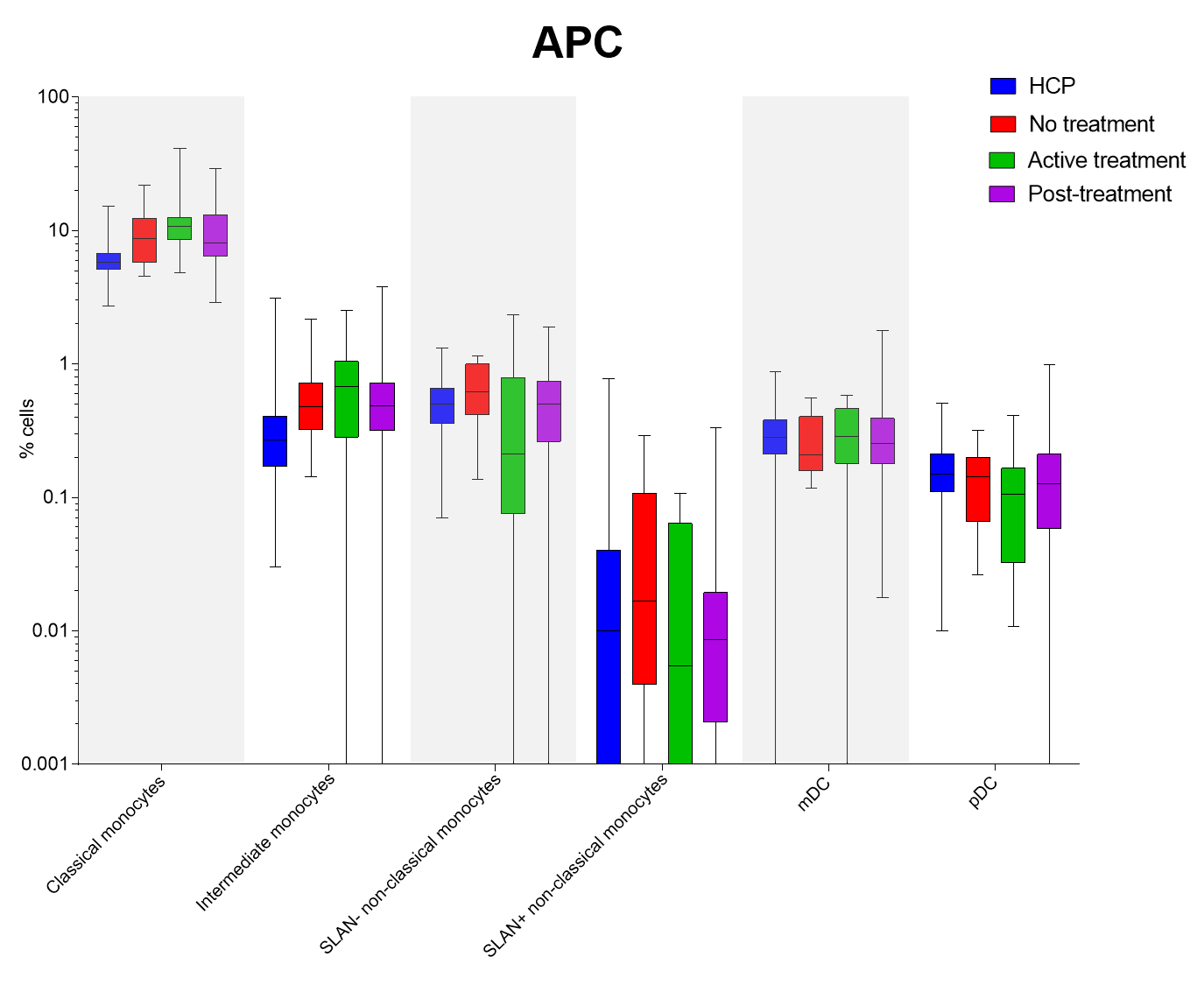


**C)** CD4 T cells


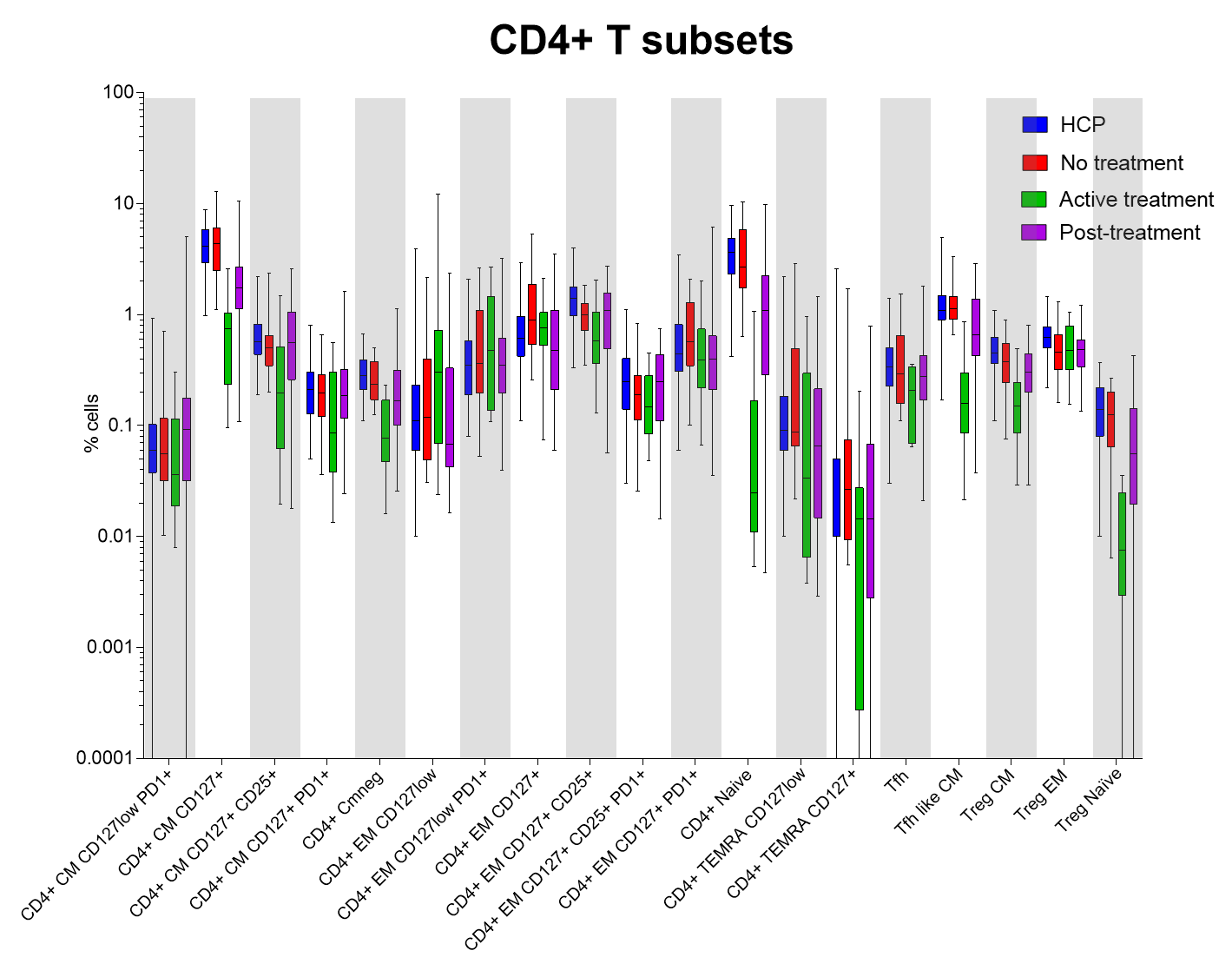


**D)** CD8 T cells


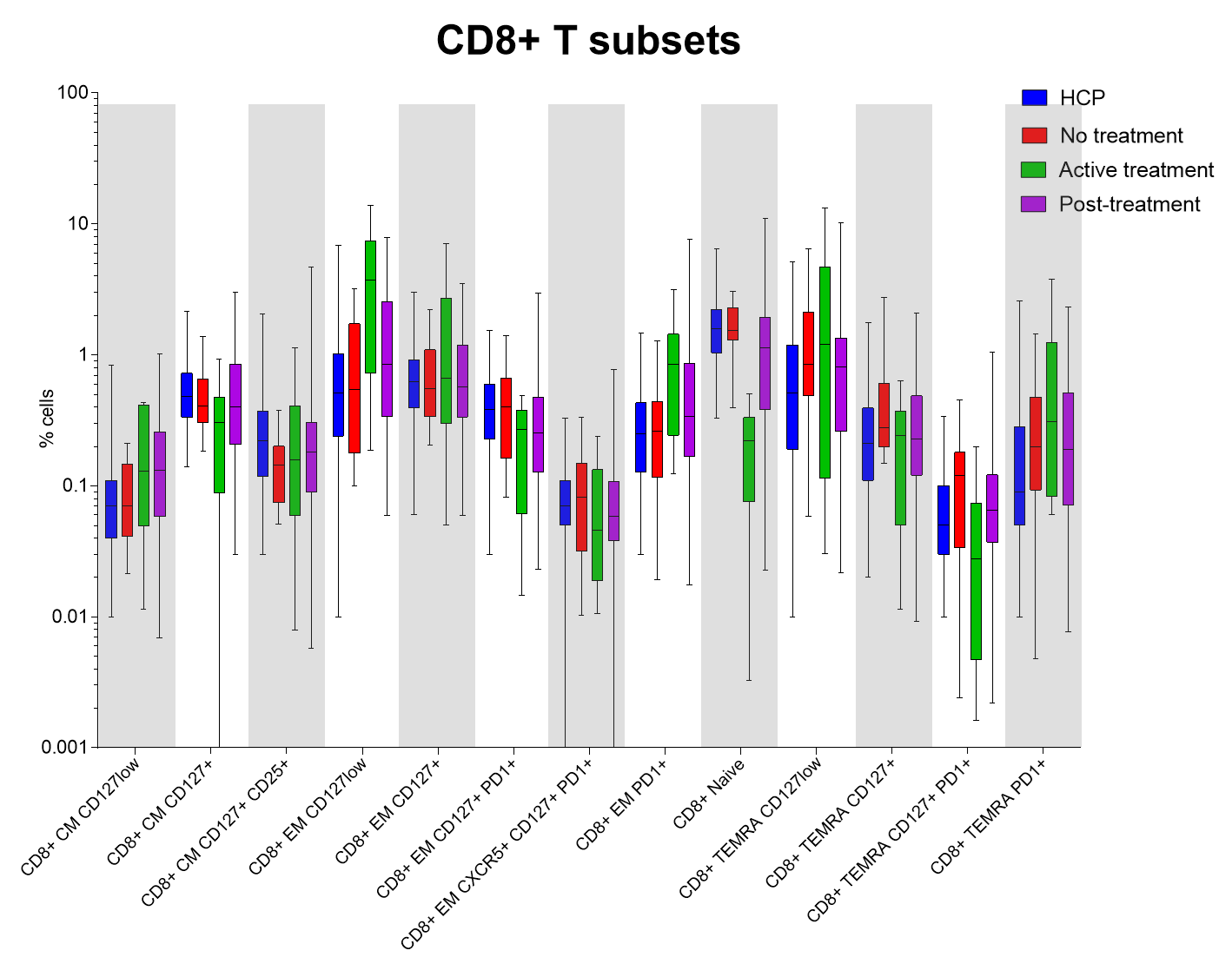


**E)** B cells


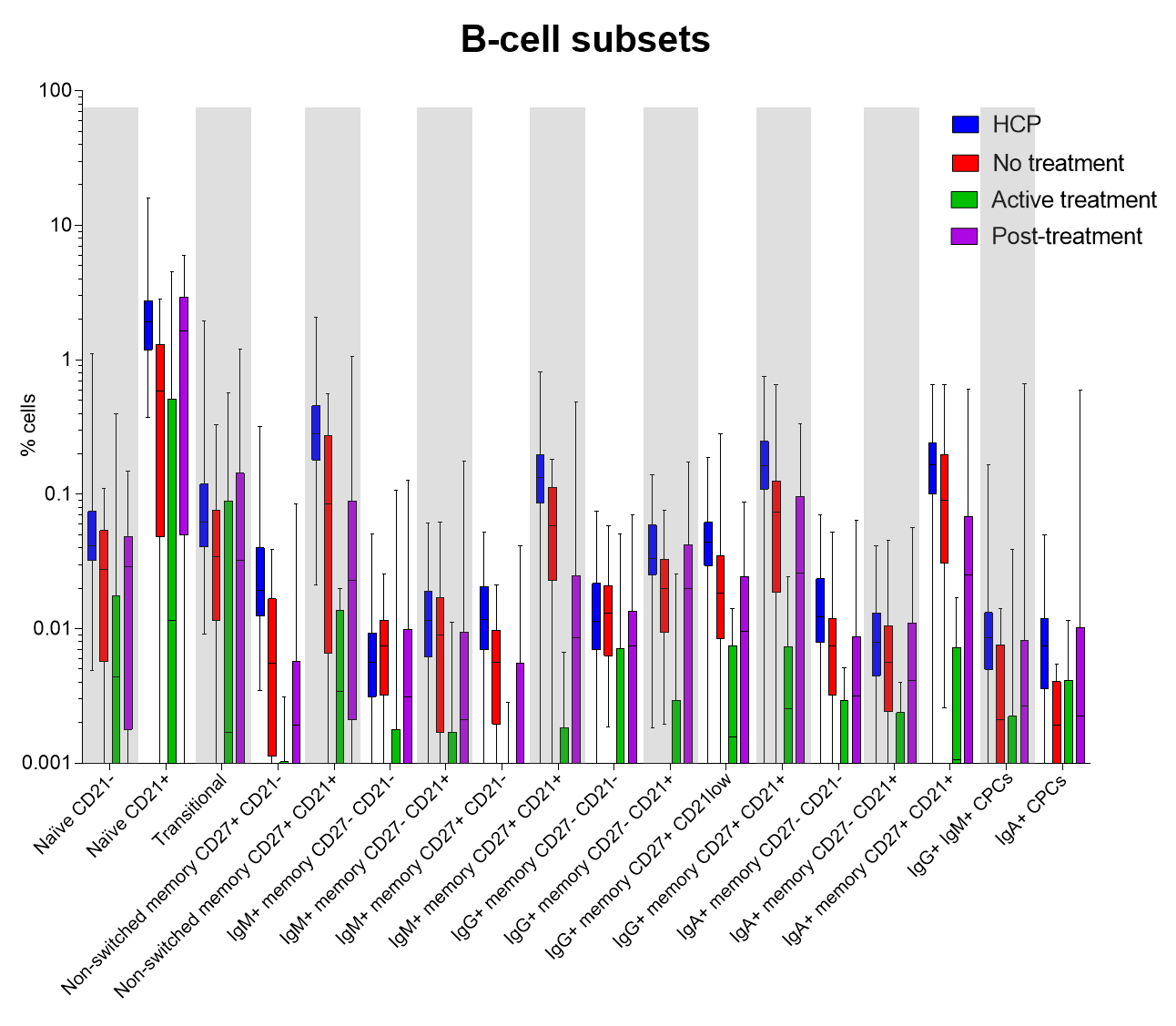


**Supplemental Figure 4.** Index of IgM, IgG and IgA antibodies against the S-glycoprotein in health care practitioners (HCP) (N = 102) and hematological patients (N = 83) after second-dose vaccination. (***, *P* < .001).

**Supplemental Figure 5. IgG levels against the receptor binding domain (RBD) in health care practitioners (HCP) and patients with hematological malignancies, after BNT162b2 or the mRNA-1273 vaccine.** Antibody levels were measured at day 7 after the second-dose vaccination in 102 HCP and 83 patients. Horizontal lines represent the median value. (*, *P* < .05).

**Supplemental Figure 6. IgG levels against the receptor binding domain (RBD) in hematological patients and health care practitioners (HCP).** The concentration of anti-RBD-IgG antibody was measured after the second-dose vaccination in 102 HCP and 83 patients. Horizontal lines represent the median value. (***, *P* < .001).

**Supplemental Figure 7.** Relative distribution of each of the 59 immune cell-types in patients with hematological malignancies, according to their clustering into the two immunotypes identified in Figure 5 of the main text. Cluster 1 refers to the largest branch, and Cluster 2 corresponds to the smallest one, with lower seroconversion rates).

**A)** Granulocytes


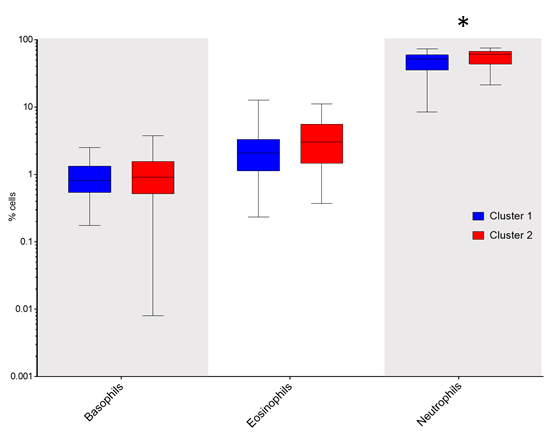


**B)** Antigen-presenting cells


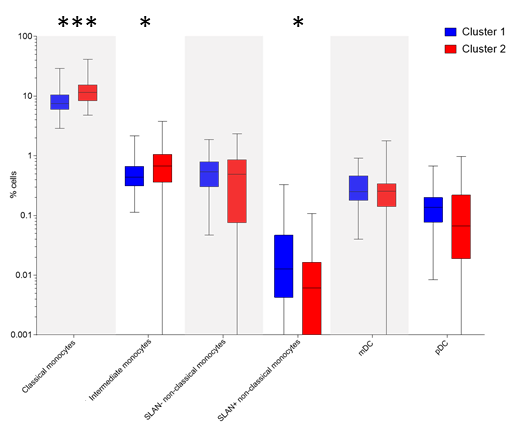


**C)** CD4 T cells


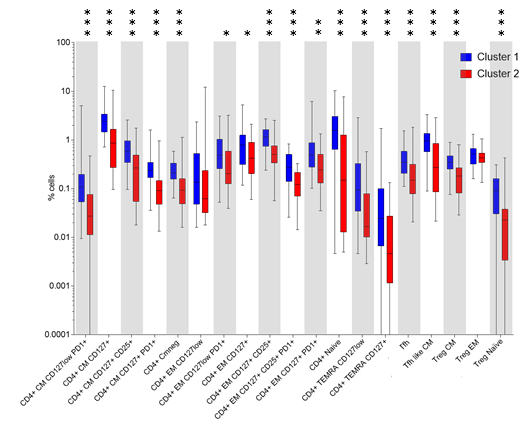


**D)** CD8 T cells


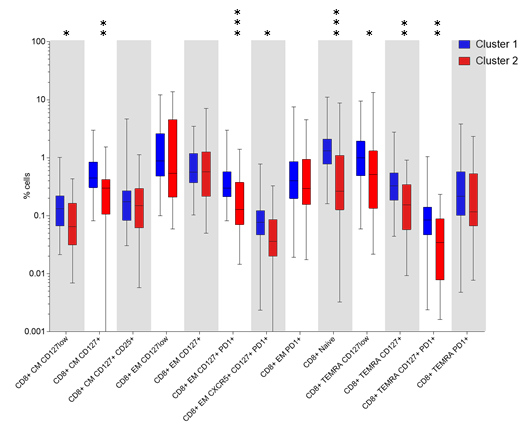


**E)** B cells


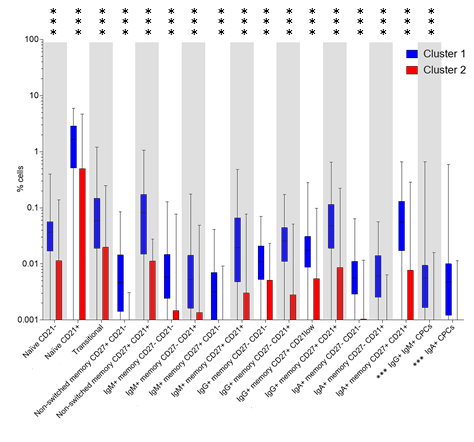

Supplement: Supplementary file 1 — Supplemental Material [file 41408_2021_594_MOESM1_ESM.docx]
